# Joint Transcriptomic and Metabolomic Analyses Reveal Changes in the Primary Metabolism and Imbalances in the Subgenome Orchestration in the Bread Wheat Molecular Response to *Fusarium graminearum*

**DOI:** 10.1534/g3.115.021550

**Published:** 2015-10-04

**Authors:** Thomas Nussbaumer, Benedikt Warth, Sapna Sharma, Christian Ametz, Christoph Bueschl, Alexandra Parich, Matthias Pfeifer, Gerald Siegwart, Barbara Steiner, Marc Lemmens, Rainer Schuhmacher, Hermann Buerstmayr, Klaus F. X. Mayer, Karl G. Kugler, Wolfgang Schweiger

**Affiliations:** * Plant Genome and Systems Biology, Helmholtz Zentrum München, Neuherberg, D-85764, Germany; † Center for Analytical Chemistry (IFA-Tulln), BOKU - University of Natural Resources and Life Sciences, Tulln, A- 3430, Austria; ‡ Institute for Biotechnology in Plant Production (IFA-Tulln), BOKU - University of Natural Resources and Life Sciences, Tulln, A-3430, Austria

**Keywords:** Fusarium head blight, resistance QTL, *Fhb1*, *Qfhs.ifa-5A*, deoxynivalenol

## Abstract

*Fusarium* head blight is a prevalent disease of bread wheat (*Triticum aestivum L*.), which leads to considerable losses in yield and quality. Quantitative resistance to the causative fungus *Fusarium graminearum* is poorly understood. We integrated transcriptomics and metabolomics data to dissect the molecular response to the fungus and its main virulence factor, the toxin deoxynivalenol in near-isogenic lines segregating for two resistance quantitative trait loci, *Fhb1* and *Qfhs.ifa-5A*. The data sets portrait rearrangements in the primary metabolism and the translational machinery to counter the fungus and the effects of the toxin and highlight distinct changes in the metabolism of glutamate in lines carrying *Qfhs.ifa-5A*. These observations are possibly due to the activity of two amino acid permeases located in the quantitative trait locus confidence interval, which may contribute to increased pathogen endurance. Mapping to the highly resolved region of *Fhb1* reduced the list of candidates to few genes that are specifically expressed in presence of the quantitative trait loci and in response to the pathogen, which include a receptor-like protein kinase, a protein kinase, and an E3 ubiquitin-protein ligase. On a genome-scale level, the individual subgenomes of hexaploid wheat contribute differentially to defense. In particular, the D subgenome exhibited a pronounced response to the pathogen and contributed significantly to the overall defense response.

Bread wheat (*Triticum aestivum*, 2n = 6x = 42, AABBDD) is one of the most important food crops worldwide, providing approximately 20% of the daily human calorie consumption (Brenchley *et al.* 2012). Increasing nutritional demands by a growing world population and environmental stresses present major challenges for wheat research and breeders. One of the most prevalent diseases on wheat and other small grain cereals is *Fusarium* head blight (FHB). The disease is mainly caused by the hemibiotrophic fungus *Fusarium graminearum*, which thrives under humid and temperate conditions, leading to large economic losses (McMullen *et al.* 2008; Pirgozliev *et al.* 2003). The most severe effect of FHB is the contamination of grains with mycotoxins such as deoxynivalenol (DON), which remain in the food chain and constitute a threat to the health of animals and humans (Pestka 2010). DON is a potent inhibitor of protein biosynthesis and, although its presence is not required to establish the infection site, it is essential for the pathogen to breach the barrier from the initially infected spikelet and its spread into the surrounding tissue (Jansen *et al.* 2005).

The wheat defense response includes a plethora of well-described mechanisms, including the biosynthesis of phenolics, polyamines, and other secondary metabolites, cell wall fortification, as well as countermeasures to reduce oxidative stress and to inactivate DON (reviewed in Kazan *et al.* 2012; Walter *et al.* 2010). Little is known on how the adaptations in the primary metabolism contribute to resistance against *F. graminearum*. Schwachtje and Baldwin (2008) discussed roles for the primary metabolism that surpass its function in nutrient acquisition for the costly defense response. These may act in defense signaling, contribute to defense by themselves, or work toward shifting recourses between infected/noninfected tissues to increase tolerance. In particular, the production of secondary metabolites is strongly linked to the expression of genes providing carbon, nitrogen, and sulfur equivalents (Aharoni and Galili 2011). Therefore, changes in the primary and secondary metabolisms need to be equally considered when observing plant/pathogen interactions.

Although bread wheat is considered susceptible to FHB, a diverse collection of resistant germplasm has been identified. More than 100 quantitative trait loci (QTL) have been described to contribute in varying extents to resistance against FHB (Buerstmayr *et al.* 2009). Yet, none of the underlying molecular mechanisms has been resolved to date. Two major and reproducible QTL derive from the Chinese spring wheat cultivar Sumai-3: *Fhb1*, located on the short arm of chromosome 3B, confers high resistance against spreading of the disease (type II) (Anderson *et al.* 2001; Buerstmayr *et al.* 2002), whereas *Qfhs.ifa-5A*, on 5AS, mainly confers resistance against initial infection (type I) (Buerstmayr *et al.* 2003).

A small number of studies investigated the differential transcriptional response to the pathogen in lines differing in the presence of *Qfhs.ifa-5A* (Kugler *et al.* 2013; Schweiger *et al.* 2013). In contrast, *Fhb1* has been investigated widely and was introduced successfully into US elite breeding material (Jin *et al.* 2013). *Fhb1*-containing lines exhibited an improved ability to transform DON into the nontoxic DON-3-glucoside (Lemmens *et al.* 2005). Still, several transcriptomic and metabolomic studies that compared lines segregating for *Fhb1* did not lead to the identification of a causal gene responsive for this mechanism (Gunnaiah *et al.* 2012; Jia *et al.* 2009; Kugler *et al.* 2013; Schweiger *et al.* 2013; Walter *et al.* 2008; Warth *et al.* 2015; Xiao *et al.* 2013; Zhuang *et al.* 2013)

A comparison of results between all these studies is challenging because they show little overlap due to the different investigated germplasms, sampling/inoculation procedures, and statistical methods used. Moreover, transcriptomic studies, including our own (Kugler *et al.* 2013; Schweiger *et al.* 2013), were long impeded by incomplete and frequently changing reference gene sets and incomplete gene annotations for bread wheat. All these factors have made it difficult to gain a complete picture of the transcriptomic response to the pathogen and to make a comparison between different studies. Recently, a comprehensive wheat survey sequence gene set has become available by the International Wheat Genome Sequencing Consortium (IWGSC) (Mayer *et al.* 2014). This reference provides a nearly complete mapping resource for transcriptomic studies. It comprises about 99,000 high-confidence genes allocated to the corresponding subgenomes and chromosome arms in version 2.2 of the annotation. To a large extent, genes were also linearly ordered (Mayer *et al.* 2009).

We have used the corresponding newly available gene models to revisit the data from Kugler *et al.* (2013), which describe the transcriptional response to *F. graminearum* in four near-isogenic lines (NILs) segregating for *Fhb1* and *Qfhs.ifa-5A*. In this study we combined a gene coexpression network with differential gene expression analysis and metabolomics measurements, which were obtained from a time-course series. We identified QTL and treatment specific network components, which aided in the reconstruction of alterations in the bread wheat primary metabolism in response to the pathogen and identified pathway components showing distinct changes for lines harboring *Qfhs.ifa-5A*. Likely candidate genes for either QTL emerged from the analysis of QTL-specific modules in our network. With the bread wheat genome sequence at hand, it is now possible to also address the subgenome-specific contributions in the pathogen response on a genome-wide scale, which highlights a prominent role of the D subgenome.

## Materials and Methods

### Plant experiments

**Figure 1:**
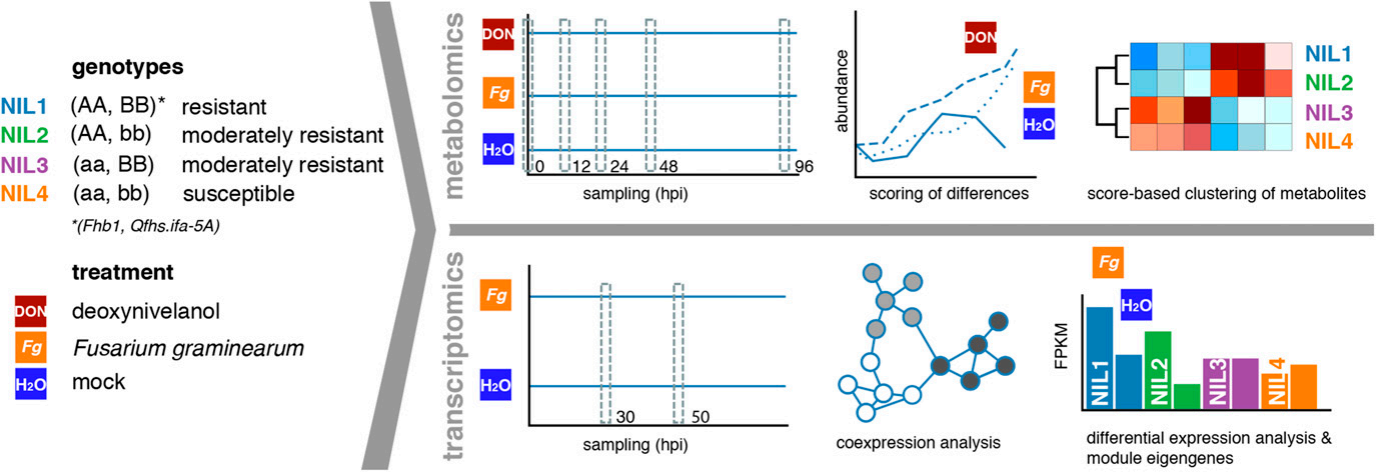
Source material, experimental conditions, and analyses. Each near-isogenic line (blue: NIL1, green: NIL2, purple: NIL3, orange: NIL4) contains either the resistant or susceptible alleles of *Fhb1* (AA, aa) and *Qfhs.ifa-5A* (BB, bb). Plants were either inoculated with *F. graminearum* spore suspensions, DON, or water as control. Samples were collected at different time points as indicated by gray, dashed boxes and subjected to RNA-sequencing (transcriptomics) or gas chromatography-mass spectrometry analysis (metabolomics). Transcriptomics data were characterized by the use of a coexpression network approach and differential expression analysis. Metabolomics data were characterized by the calculation of a Bayes factor score and clustering of these scores.

The procedures to capture the metabolomics data (Warth *et al.* 2015) and the transcriptomics data (Kugler *et al.* 2013) used similar plant material, growth conditions, and inoculation and sampling procedures with *F. graminearum* spore suspensions or DON (metabolomics dataset only), which also were described in the respective references. The metabolomics data set generated from *F. graminearum*−inoculated plants comprises novel data generated similarly as described in Warth *et al.* (2015) for the DON-treated samples. The employed BC5F2 NILs have the susceptible German spring wheat cultivar Remus as the recurrent parent. They harbor both (NIL1), either (NIL2: *Fhb1*, NIL3: Qfhs.ifa-5A), or neither of the two resistance QTL, which were introgressed from CM-82036, a Mexican spring wheat line. Metabolomics samples were taken at time points 0, 12, 24, 48, and 96 hours past infection/inoculation (hpi). Transcriptomics samples were taken at 30 and 50 hpi (Figure 1).

The metabolomics experiments have been conducted in a light- and temperature-controlled greenhouse in spring 2012 in full compliance with the Metabolomics Standards Initiative (Sumner *et al.* 2007). The transcriptomics experiment was conducted under comparable controlled light and climatic conditions in a growth chamber because of limited greenhouse space in Fall 2011. In the transcriptomics experiment 12 florets per head (the two basal florets of six central spikelets) were inoculated at anthesis with 10 μL of a *F. graminearum* conidia spore suspension (strain IFA65, concentration 50,000 conidia/mL) or mock by cautiously inserting a droplet onto the generative part of each floret. Similarly, for the metabolomics experiment 20 florets per head (from 10 central spikeles) were inoculated to yield sufficient tissue for analysis with either 10 µL of a *F. graminearum* conidia spore suspension (10,000 macroconidia/mL), DON (5 g/L in water), or mock. The treated heads were moistened and covered in plastic bags for 24 hr to provide humid conditions for the pathogen. Only palea and lemma of the inoculated florets were sampled, including the respective part of the rachis at the indicated time points and shock frozen in liquid nitrogen.

### Metabolite analysis

After milling, extraction, and evaporation of the samples, an online two-step derivatization procedure was performed with the use of methoxyamine hydrochloride and *N*-methyl-*N*-trimethylsilyl trifluoroacetamide. Analytes were separated and detected on an Agilent 7890A gas chromatograph coupled to a 5975C inert XL MSD detector (Agilent, Waldbronn, Germany). Raw data were processed with the MetaboliteDetector software (Hiller *et al.* 2009). Gas chromatography with electron impact mass spectrometry spectra and retention indices of recognized features were compared with an in-house library, which was established with commercially available reference standards. Hence, most metabolites reported herein can be regarded as “level 1 – identified compounds” (Sumner *et al.* 2007). Chromatography and mass spectrometry features for the 59 identified metabolites as well as information on the identification level can be found in supporting information, Table S1. Data normalization was performed before statistical analysis with wheat aggregate QC samples and the internal standard nonadecanoic acid methylester. Missing value imputation and outlier testing was performed by tailored in-house R scripts (Warth *et al.* 2015). A multivariate evaluation of the DON-treated and mock samples (Warth *et al.* 2015) and the corresponding metadata is publically available via the MetaboLights metabolomics data repository (Salek *et al.* 2013) (accession number MTBLS112). The metadata for *F. graminearum* infected samples also is provided through the MetaboLights database (accession number MTBLS153). In Stegle *et al.* (2010) a Gaussian process (GP) based on two conditions test (GP2S) was introduced for detecting differential expression between two conditions. We applied GP2S tests on the metabolite time series data for a pair-wise comparison of treatment effects. The GP2S test compares two models: The first model assumes that the time series measurement in both conditions control and treatment are samples drawn from a shared distribution. The alternative model describes the time series in both conditions as sampled from two independent distributions. We marked scores larger than log(3)=1.099 as indications for substantial differences in treatments.

### Gene expression analysis

Extracted RNA from sampled tissues was sequenced on an Illumina HiSeq2000 by an external sequencing provider (GATC, Konstanz, Germany). A detailed description of the generation of the RNA-seq data are given in Kugler *et al.* (2013). The corresponding data can be downloaded from Arrayexpress database under accession number E-MTAB-1729 (Kolesnikov *et al.* 2014).

A total of 1800 million single-end reads were mapped against the IWGSC bread wheat reference assembly. One replicate was removed after quality control (NIL2, M50, replicate 3). Reads were mapped against the reference by the use of TopHat (Trapnell *et al.* 2012) and Bowtie (Langmead and Salzberg 2012) with default parameters and keeping only unambiguously matched reads (Table S2). Mapped reads were transformed to FPKM values and normalized using Cufflinks (Trapnell *et al.* 2012). To test for differential expression [false-discovery rate (FDR)-adjusted *P* < 0.1, absolute log2 fold change >1], we applied the edgeR package in R (Robinson *et al.* 2010) on the raw counts as extracted with HTSeq (Anders *et al.* 2015) for the IWGSC high confidence genes. We tested mock-treated samples against *F. graminearum*-inoculated samples for all four NILs at both time points (Table S3) and for differences between NIL4 and the other NILs (Table S4). Deviations from the expected A, B, and D subgenome distribution (Mayer *et al.* 2014) were assessed with χ^2^ goodness-of-fit tests against 10,000 random multinomial distributions. A gene coexpression network comprising 18,948 genes was constructed using the weighted correlation network analysis (WGCNA) method (Langfelder and Horvath 2008), after a pseudocount transformation l2FPKM=(log2(FPKM+1)), a coefficient of variation filter (CV > 0.4), and keeping genes that surpassed a minimum expression level defined by the 5% percentile of all expressed genes The model was fit to a power law distribution (network type unsigned; power = 11), and the data clustered based on the Topological Overlap Matrix (Langfelder *et al.* 2008) using the cutreeDynamic method (minClusterSize = 50; deepSplit = 2; pamRespectsDendro = FALSE, merging close modules at 0.9; Figure S1). Intramodular hub genes were defined by the top 10% percentile of the intramodular connectivity. Eigengenes were calculated using the WGCNA package (Langfelder and Horvath 2008).

Enrichment of Gene Ontology (GO) terms was assessed with the Bioconductor package GOstats using conditional hypergeometric tests (Falcon and Gentleman 2007). Key enzymes were extracted by using their *A. thaliana* counterparts (Table S5) and sequence homology searches based on blastp (Altschul *et al.* 1990) with at least 60% sequence coverage. The 8605 gene triplets were based on a reciprocal best hit criterion in pairwise subgenome matches similar to the approaches in Pfeifer *et al.* (2014) and Mayer *et al.* (2014) and applying an identity threshold of 90%. For the gene triplet expression analysis gene triplets were concatenated into a triplet matrix as described in Mayer *et al.* (2014) and Pfeifer *et al.* (2014). The triplet coexpression network was inferred analogical to the overall network (CV threshold 0.7; lower expression 10% percentile; network type “signed hybrid”, beta = 8; minimal module size= 20; no merging of modules; Figure S2). The significance of differences in the subgenome-wise expression were quantified with a Kruskal-Wallis test and subsequent pair-wise Mann-Whitney *U* tests, as followed by an FDR adjustment of *P* values.

### Data availability

Data availability is as follows: RNA-seq: EBI Arrayexpress (E-MTAB-1729); gas chromatography–mass spectrometry: MetaboLights (MTBLS112, MTBLS153).

## Results

### Integrated data analysis of the wheat response against *F. graminearum* and DON

The aim of this study was to explore QTL-associated differences in the response of four bread wheat NILs (NIL1−4) after inoculation with *F. graminearum* or DON by combining gene expression and metabolomics measurements (Figure 1). The investigated four BC5F2 NILs share the common susceptible genetic background of the recurrent parent Remus but are different for introgressed resistance QTL *Fhb1* and *Qfhs.ifa-5A* from the donor line CM-82036, providing them with distinct resistance levels (Schweiger *et al.* 2013). To describe differences in transcript abundances, we used the recent IWGSC bread wheat genome sequence assembly (Mayer *et al.* 2014) as a mapping reference for RNA-seq measurements (Kugler *et al.* 2013). The RNA-seq data comprised samples from two time points, 30 and 50 hpi, with either *F. graminearum* spore suspensions or mock treatment. To complement expression profiles with functional output, we integrated gas chromatography-mass spectrometry−derived metabolite measurements from a dense time course of similar inoculation experiments on these NILs with the fungus and additionally with its major toxin DON (Warth *et al.* 2015).

**Figure 2:**
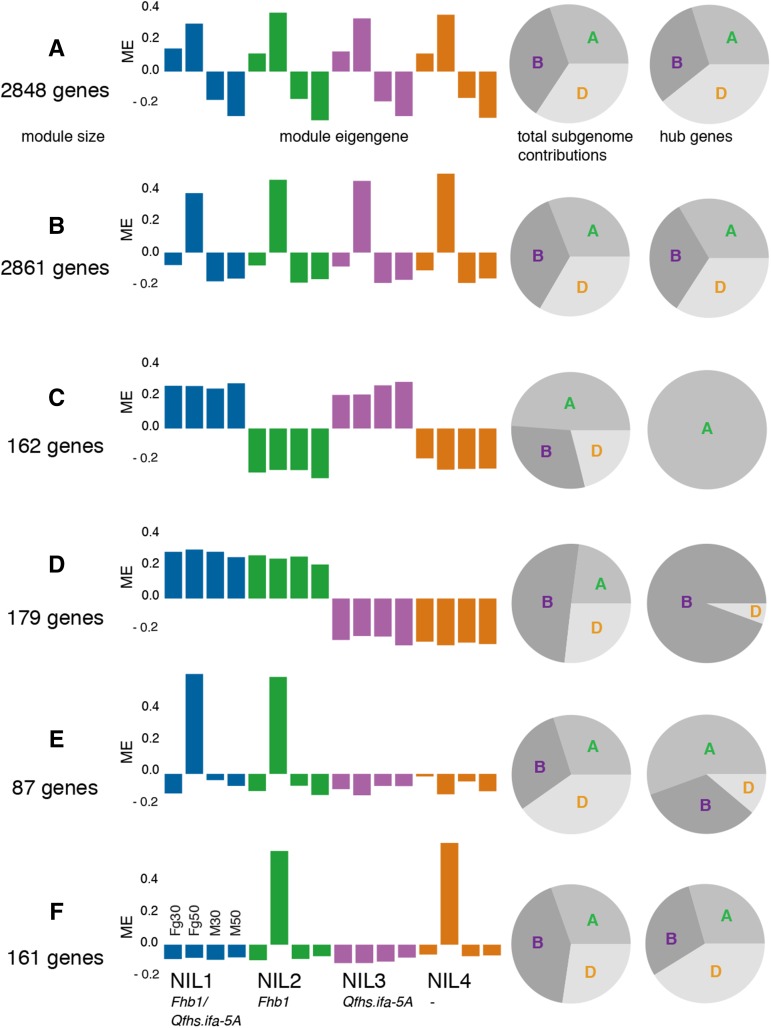
Coexpression network modules. RNA-seq data were clustered into modules by inferring a weighted coexpression network. (A−F) Selected modules characterized by a general response to the fungus or are specific for QTL. The module eigengene panels (left) summarize the module-wise expression (Fg: treatment with *F. graminearum*, M: mock treatment, 30: 30 hpi, 50: 50 hpi; blue: NIL1, green: NIL2, purple: NIL3, orange: NIL4). Pie charts give the ratios of genes contributed by the individual subgenomes for the entire module (left) and for intramodular hub genes (right).

To gain insights into system-wide expression patterns we first used the WGCNA approach (Langfelder and Horvath 2008) for grouping genes into sets of similar expression patterns. Genes within these groups show strongly correlated expression, which indicates common regulatory mechanisms or concerted actions. The distinct expression profiles of modules are represented by the module eigengene (Langfelder and Horvath 2007), which correspond to the first principal component of the module expression matrix and which can be regarded as a representative for the gene expression in a module. Putative functional characterizations of modules regarding *F. graminearum*-resistance and QTL activity were derived by integration of differential expression information, GO enrichments, and the corresponding module eigengene. Additionally, by quantifying intramodular connectivity, we also extracted hub genes for each module. On the basis of their expression profiles and their functional characteristics, we selected six network modules to be of special interest for subsequent investigations (Figure 2). Processed transcriptomic data and analysis results described in this study are available at the project’s RNASeqExpressionBrowser (Nussbaumer *et al.* 2014a) Web site (http://pgsb.helmholtz-muenchen.de/cgi-bin/db2/BOKUnils/index.cgi). The metabolomic data set for DON-treated NILs (Warth *et al.* 2015) and *F. graminearum*-inoculated NILs comprised 59 metabolites mainly derived from the primary metabolism sampled at 0, 12, 24, 48, and 96 hpi (Table S6). The differential abundance of metabolites among DON, *F. graminearum*, and mock treatment was quantified by the use of a Gaussian processes-based score, which was calculated for each metabolite reflecting differences between two conditions in metabolite abundance over time (Stegle *et al.* 2010). The scoring compares two alternative models in which the data from the two conditions is either explained by one single (shared) process or by two independent processes (one for each condition). The two alternatives (shared or independent model) can then be compared, with larger scores indicating more pronounced differences between the conditions (Table S7).

### Increased turnover rates in a restructured primary metabolism fuel a broad defense response

We first investigated the coexpression network for gene functions in response to the pathogen by looking into functional enrichments and characteristic expression patterns. The two largest modules of the coexpression network, module A (2848 genes) and module B (2861 genes), grouped genes with strong general responses to the pathogen. Both were highly enriched for *F. graminearum*-responsive genes across all NILs regardless of individual resistance levels (one-sided Fisher’s test; FDR-adjusted *P* < 0.05; Figure 2, A and B and Figure S3). Genes in module A were differentially expressed in *F. graminearum*-inoculated samples at 30 hpi and 50 hpi (Figure 2A), whereas genes in module B were more specific for 50 hpi (Figure 2B). Although the subgenome distribution for all genes in these two modules showed no obvious bias, the distribution of the hub genes showed a slight overrepresentation of genes from the D subgenome.

Overrepresented GO terms in module A reflected a broad response to the pathogen. These comprise functions in signaling, the defense against oxidative stress, the biosynthesis of tryptophan, and defense-associated secondary metabolites such as phenylpropanoids. Moreover this group included chitinases, proteinase inhibitors, and efflux pumps (Table S8). Several GO terms corresponded to the primary metabolism, which include a strongly up-regulated sucrose-phosphatase (*Traes_1DS_9AE5A76AC* in GO:0009312) and genes involved in the biosynthesis of thiamin (GO:0009229). Sucrose phosphatases mediate the last step in the biosynthesis of sucrose, the plants main transport form of carbohydrates. We also found elevated sucrose levels in our metabolomics experiment although only for DON-treated samples (Figure S4 and Figure S5). Abundant sucrose is broken down to fuel the tricarboxylic acid cycle (TCA) predominantly via glycolysis. The increased biosynthesis of thiamin relates to key enzymes in glycolysis and the TCA, pyruvate dehydrogenase and 2-oxoglutarate dehydrogenase, which require thiamine diphosphate as cofactor.

**Figure 3:**
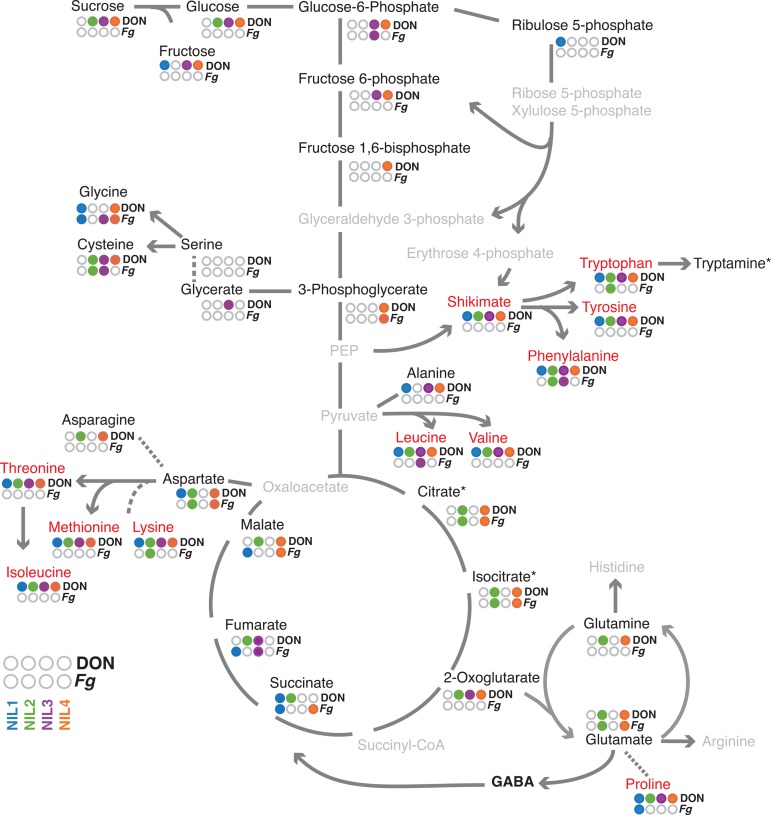
Changes in the primary metabolism in response to *F. graminearum* and DON. Metabolites quantified by gas chromatography-mass spectrometry are set in black, and nonmeasured and nondetected metabolites are set in gray. Treatment-responsive metabolites (DON and *F. graminearum* (Fg); Table S2) in the individual lines are indicated by color (blue: NIL1, green: NIL2, purple: NIL3, orange: NIL4). Levels of metabolites set in red were strongly changed in response to DON or *F. graminearum* (average score greater than 10). Genes with significantly changed transcript abundances are indicated by arrows/lines set in gray, whereas dashed lines indicate no significantly changed transcript in any line. *Citrate and *isocitrate could not be distinguished due to chromatographic coelution. *Tryptamine levels were at or below the methods detection limit in most samples and thus could not be safely quantified.

To further expand on these observations we evaluated changes in transcript and metabolite abundances associated with *F. graminearum*/DON treatments in the respiratory chain and in the metabolism of glutamate. We extracted the expression profiles of all genes encoding for respective protein functions and generated eigengenes for each group of genes as the representative expression value. The expression of genes in glycolysis and the TCA cycle was strongly associated with *F. graminearum*−inoculated samples (Figure 3 and Table S9). In addition, the expression of genes encoding key enzymes in the pentose-phosphate pathway, glucose-6-phosphate dehydrogenase, and gluconate-6-phosphate dehydrogenase were strongly linked to pathogen treatment. The pentose-phosphate pathway provides an alternative route for the breakdown of hexoses into glycerinaldehyde-3-phosphate. It also generates nicotinamide adenine dinucleotide phosphate and erythrose-4-phosphate required in the shikimate pathway and ultimately for the production of phenylpropanoids. Despite increased abundances in transcript levels the corresponding metabolite levels in the glycolysis and pentose-phosphate pathway remain mostly unchanged (Figure 3, Figure S4, and Figure S5).

### Differences for Qfhs.ifa-5A in the TCA and the metabolism of glutamate

**Figure 4:**
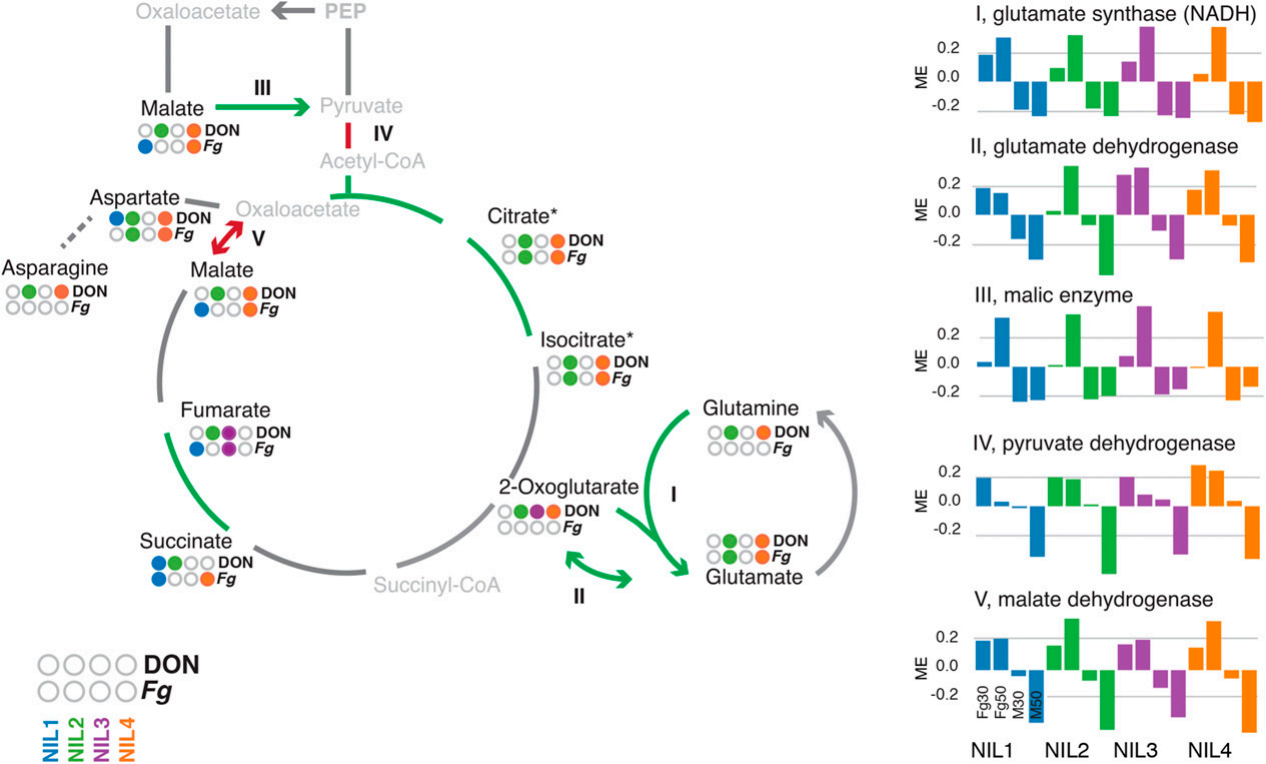
QTL-associated differential transcript and metabolite abundances in the glutamate metabolism. Metabolites quantified by GC-MS are set in black, and nonmeasured and nondetected metabolites are set in gray. Treatment responsive (DON and *F. graminearum* (Fg); l Table S2) metabolites in the individual lines are indicated by color (blue: NIL1, green: NIL2, purple: NIL3, orange: NIL4). Green arrows/lines highlight genes with increased expression at 30 hpi for *Qfhs.ifa-5A*, red lines represents decreased expression at 50 hpi for *Qfhs.ifa-5A*. These expression differences are visualized for the isogene families by eigengene values (I-V). The individual NILs are distinguished by color. The four bars per line represent *F. graminearum*−inoculated samples at 30 and 50 hpi and mock-treated samples at 30 and 50 hpi from left to right.

The metabolism of glutamate as a means to procure nitrogen for the biosynthesis of amines including amino acids depends on the availability of the TCA intermediate 2-oxoglutarate, which serves as a link to the TCA cycle. Genes of the TCA cycle and the metabolism of glutamate were in general responsive to the pathogen, yet distinct differences existed for lines harboring *Qfhs.ifa-5A*−related differences (Figure 4): Ammonia assimilation is mediated by glutamine synthetases (GS) and glutamate synthases (GOGAT), which generate glutamate from 2-oxoglutarate via glutamine. In plants the cytosolic (GS1) and the chloroplastic (GS2) isoenzymes for GS are regulated differentially with respect to tissue and external stimuli, assuring timely acquisition of ammonium from different sources (Miflin and Habash 2002). Three cytosolic GS1 genes showed increased transcript abundances in response to the pathogen and also exhibited by far the greatest expression rates as compared with 11 remaining wheat GS genes (Figure S6). For the GOGAT, we found a similar response for five NADH-dependent isozymes but not for cytosolic ferredoxin-dependent GOGAT (I in Figure 4). The expression of these genes was more strongly associated to the earlier infection time point 30 hpi in lines containing *Qfhs.ifa-5A*. A closer inspection of interrelated genes showed greater transcript abundances for many of them at 30 hpi for *Qfhs.ifa-5A*: This included most of the genes encoding TCA cycle steps as well as glutamate dehydrogenases and malic enzymes (II and III in Figure 4 and Table S9). Cytosolic malic enzymes provide additional pyruvate to the TCA cycle. The required malate originates from oxaloacetate, which most likely stems from the also strongly *F. graminearum*−responsive phosphoenolpyruvate carboxylase (Table S6 and Table S9). Yet, the largest QTL-effects were observed for pyruvate dehydrogenases and malate dehydrogenases (IV and V in Figure 4), which did not exhibit earlier expression for the QTL but showed strongly reduced expression levels at 50 hpi in lines harboring *Qfhs.ifa-5A*. Abundance pattern of metabolite intermediates for these pathways reflected the observed differences in expression levels: Glutamate and aspartate levels were changed after DON and *F. graminearum* treatment only for lines without the QTL (NIL2 and NIL4), whereas glutamine and asparagine levels in these lines were only changed in response to DON (Figure 3 and Figure 4). Within the TCA cycle malate levels (in response to DON) and citrate/isocitrate levels were similarly affected (Figure 4).

### Protein ubiquitination, elevated levels of tRNA ligases and amino acids characterize the specific response to the ribotoxic effect of DON

Module B was specific for genes expressed 50 hpi with the pathogen (Figure 2B). Independent studies demonstrated that at this time point the fungus has switched from the initial biotrophic growth to the formation of infection hyphae (Pritsch *et al.* 2000) and started to produce greater levels of DON (Audenaert *et al.* 2013). DON inhibits protein biosynthesis by interaction with the ribosomal 60S subunit and induces apoptosis via a mitogen-activated protein kinase-activated ribotoxic stress response (Pestka 2010). Accordingly, GO terms in module B were significantly enriched for terms relating to “oxidation reduction” (GO:0006979) and “translation” (GO:0006412) (l Table S8). The latter predominantly included genes encoding ubiquitin-60S ribosomal protein L40 fusion proteins. These fusion proteins act in ribosomal assembly and free ubiquitin acts in targeting nonfunctional proteins and unfinished peptide chains to the proteasome (Finley *et al.* 1989). Both mechanisms may be an active response to the effects of DON. Highly enriched genes within this module encoded for translation initiation factors (GO:0006413) and tRNA ligases (*i.e.*, GO:0043039, GO:0034660), which mediate the transfer of amino acids to the expanding peptide chain in translation. All tRNA ligases showed strong associations to *F. graminearum*-inoculated samples with the exception of glycine tRNA ligases where none of the encoding genes were greater expressed in response to the pathogen (Table S6 and Table S9). We recorded high scores for most proteinogenic amino acids in the DON/mock comparison indicating higher abundances in the DON-inoculated samples, which has been a major finding in our previous analysis (Warth *et al.* 2015). Several proteinogenic amino acids were also found changed in *F. graminearum*-inoculated samples albeit less pronounced (Figure 3, Figure S4, and Figure S5). These elevated levels derive from increased biosynthesis as reflected by the increased abundance of transcripts corresponding to most key amino acid biosynthesis genes (Figure 3, Table S6, and Table S9) except for asparagine synthase (asparagine), pyrroline-5- carboxylate reductase (proline), 3-phosphoserine phosphatase (serine) and dihydrodipicolinate synthase (lysine).

### QTL-specific modules and candidate genes emerging from the coexpression network

We identified several modules specific for either QTL: Modules C (162 genes, *Qfhs.ifa-5A*) and D (179 genes, *Fhb1*) showed significant expression differences between lines differing in the respective QTL (Figure 2, C and D). A chromoWIZ analysis showed that both modules were enriched for genes located on chromosomal regions harboring the respective QTL (Figure S7; Nussbaumer *et al.* 2014b). Hub genes were almost entirely located on the A and the B subgenomes harboring the respective QTL (Figure 2, C and D).

Hub gene expression profiles in module D characterized constitutively expressed genes for *Fhb1*-containing lines regardless of treatment or time point. Among the greatest expressed genes mapping to chromosome 3B are several protein kinases (*Traes_3B_9985A569B, Traes_3B_ADCF93AE0, Traes_3B_E81A8FACB*), a leucine-rich receptor protein (*Traes_3B_CE31EE51C*) and beta-fructofuranosidase (*Traes_3B_61E72DF24*), and a gene encoding an unknown protein (*Traes_3B_908129DB2*). Additionally, *Traes_3DS_2BD8DC857*, encoding an F-box protein, did not map to chromosome 3B of the FHB susceptible reference cultivar Chinese Spring (Figure S8).

**Figure 5:**
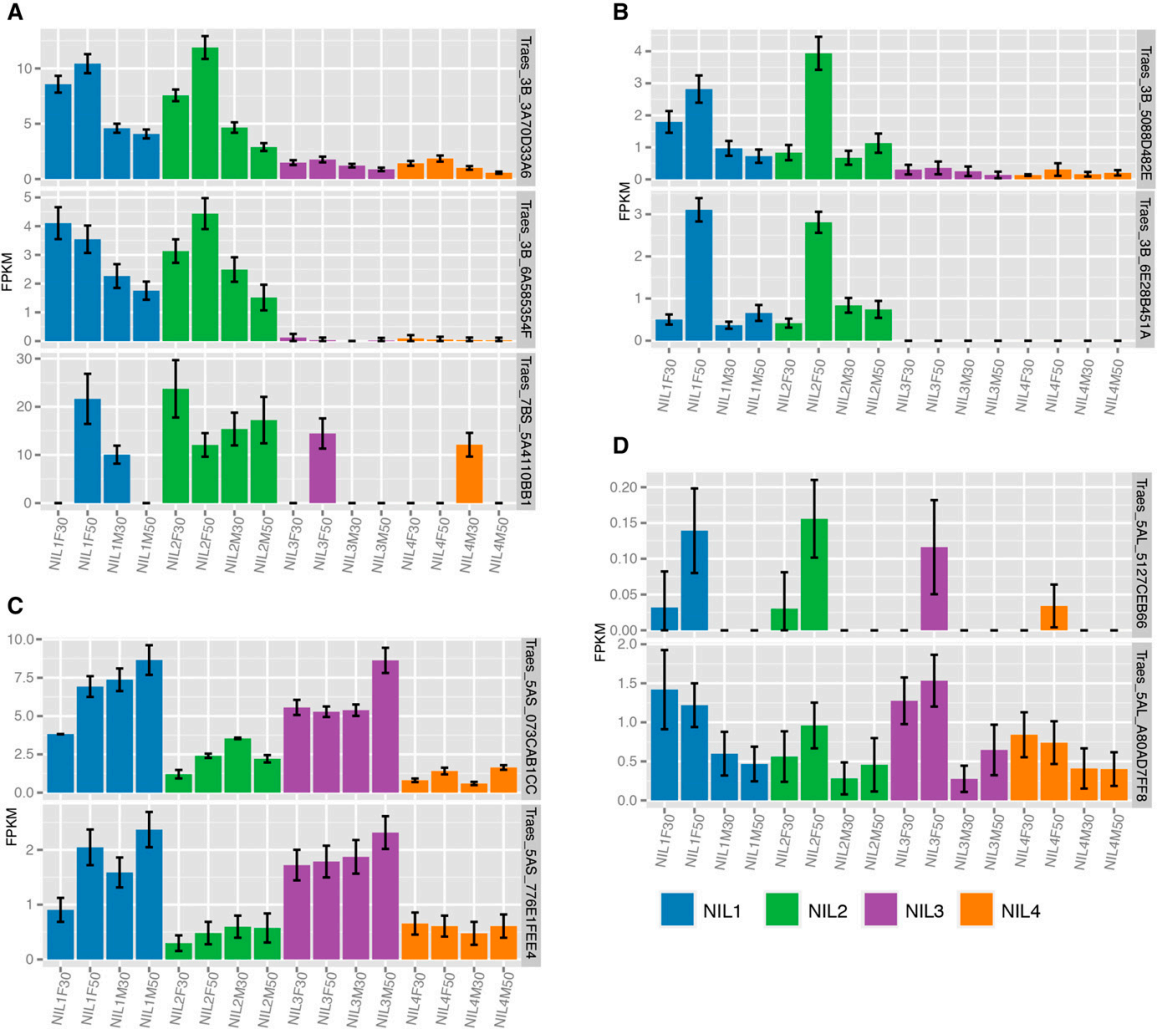
Gene expression profiles of QTL candidates. (A) *Fhb1*-associated and *F. graminearum*−responsive genes in module D; (B) *Fhb1*-associated and *F. graminearum*-responsive genes mapped to chromosome 3B in module E; (C) *Qfhs.ifa-5A*−associated constitutively expressed amino acid permeases in module C and (D) *Qfhs.ifa-5A*−associated, *F. graminearum*−responsive genes. Means of FPKM values are given for each tested experimental condition (NIL1-4, F ... *F. graminearum*−inoculated, M ... mock-treated, 30/50 ... 30/50 hai).

Several genes in module D also showed differential expression in response to *F. graminearum*: *Traes_3B_3A70D33A6*, a receptor-like protein kinase, and *Traes_3B_6A585354F*, a protein kinase, were significantly changed in response to the pathogen at 50 hpi and thus should also be considered likely candidates. *Traes_7BS_5A4110BB1*, a highly expressed and *Fhb1*-specific MATE-efflux pump, maps to chromosome 7B and could be a likely downstream target of *Fhb1*-activity (Figure 5A).

A second *Fhb1*-related module E (87 genes) included genes greater expressed in *Fhb1* lines at 50 hpi after inoculation with the pathogen (Figure 2E). Most genes within module E exhibited low transcript abundances, and we found no enrichment for 3B-mapped genes. Potentially, this module includes downstream targets of the QTL activity. Two genes emerge from this list as they were more abundant in response to the pathogen and mapped onto 3BS: *Traes_3B_5088D482E* a SINA-like 11 E3 ubiquitin-protein ligase, and *Traes_3B_6E28B451A* (unknown protein, Figure 5B). Of these, *Traes_3B_5088D482E* is the only gene in our analysis, differentially expressed also for 30 hpi in *Fhb1* containing lines. GO enrichments for either module D or module E did not allow deducing biological functions of *Fhb1*-related downstream targets.

Module C includes genes with significant expression differences between lines differing in *Qfhs.ifa-5A* (NIL1 and NIL3 compared with NIL4). In contrast to genes in module D, none of the 162 genes within module C were also changed for *F. graminearum* treatment. The set of 5A-mapped genes therein shows a constitutive expression pattern for *Qfhs.ifa-5A* and contains 50 genes (Table S10). Among these we identified two amino acid permeases (AAP, isoforms 8, *Traes_5AS_073CAB1CC* and 6, *Traes_5AS_776E1FEE4*, Figure 5C). However, *Traes_5AS_776E1FEE4* showed overall low expression levels.

Our network contained no module for genes with pathogen-specific expression changes for *Qfhs.ifa-5A* similar to module E for *Fhb1*, which matches previous observations suggesting a constitutive mode of action for the QTL (Kugler *et al.* 2013; Schweiger *et al.* 2013). Potentially, QTL-associated and pathogen-responsive genes also could be too small in numbers to form a module by themselves. Consequently, such genes could have been included in one of the pathogen-responsive modules. Two genes were significantly changed for *Qfhs.ifa-5A* and *F. graminearum*-treatment: *Traes_5AL_5127CEB66* module B, flavin-containing monooxygenase) and *Traes_5AL_A80AD7FF8* (module A, calmodulin-binding protein, Figure 5D). Alternatively, a susceptibility factor could be encoded in non-*Qfhs.ifa-5A* lines. Such genes and the downstream targets of such a factor might be included in module F (161 genes), specific for *F. graminearum*-inoculated NIL2 and NIL4 samples at 50 hpi (Figure 2F).

### A pronounced role of the D subgenome in the response to *F. graminearum*

Two hybridization steps have resulted in three homoealleles for many of the functional genes in allohexaploid bread wheat (Marcussen *et al.* 2014). The consequent functional plasticity enabled reprogramming the individual subgenomes contributions to best meet environmental challenges including the response to pathogens (Feldman and Levy 2012). We hypothesized that redundant functions within homeoalleles are regulated differentially also in response to pathogen attack. Copies from single subgenomes may contribute in an additive manner, may have diverging expression patterns, have either been shut down, giving rise to expression dominance from one or two subgenomes, or alternatively been subjected to subfunctionalization. We observed an unbalanced genome-wide distribution of *F. graminearum*−responsive greater expressed genes. Significantly more genes than expected were differentially expressed in the D subgenome and fewer in the A subgenome (Figure S9).

After these observations, we hypothesized redundant functions within homeoalleles are regulated differentially. To inspect differential contributions to a putative similar functionality by subgenomes, we employed a set of 8605 homeologous gene triplets (25,815 genes). Triplet genes are genes with a mutual best match to genes in the other two subgenomes. They have been used previously for characterizing gene expression bias in different wheat organs and the developing endosperm (Mayer *et al.* 2014; Pfeifer *et al.* 2014). In our data 1384 (16%) triplets had at least one member residing in the coexpression network. Depending on the genotype, up to 15% of all triplets included at least one pathogen-responsive differentially expressed gene at 50 hpi. Only for 25% of these triplets all three copies were differentially expressed (Figure S10A), suggesting a tight regulation of the resource-intensive defense response. All subgenomes contributed equally to the number of differentially expressed genes in triplets with only one pathogen-responsive member at 30 hpi (Figure S10B). At 50 hpi, we found a significant deviation from the expected distribution for lines lacking *Qfhs.ifa-5A* (NIL 2 and NIL 4). Here the contribution of subgenome A dominated over contributions from subgenomes B and D.

**Figure 6:**
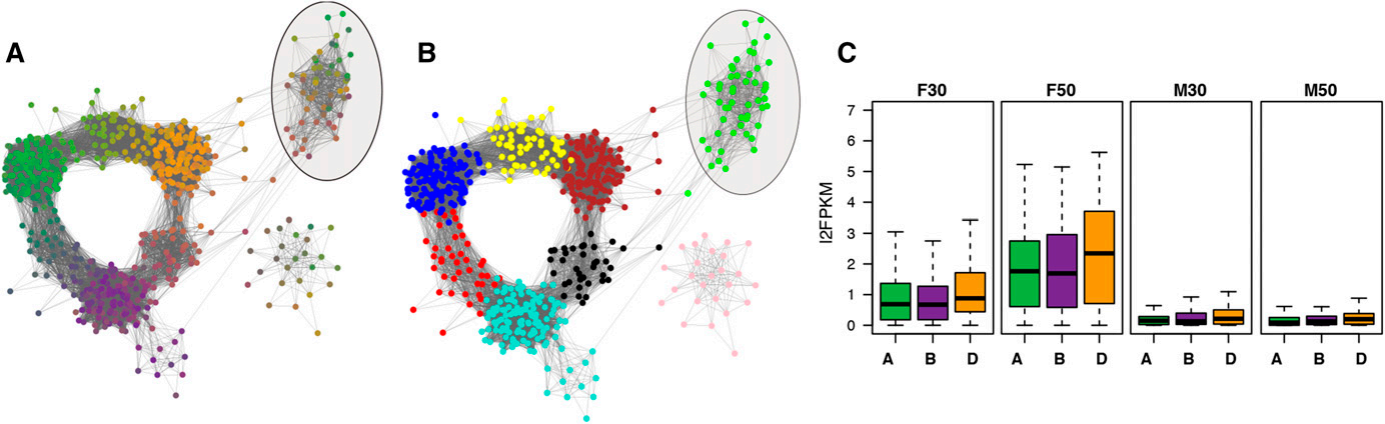
Coexpression analysis of homeologous triplet genes. A set of conserved triplet genes, with one copy per subgenome (A, B, and D) was used to investigate genome-specific expression behavior and dosage effects in a triplet-based coexpression network. (A) Coloring the network nodes by expression contributions from individual genomes highlighted regions where the combined triplet expression is dominated by a single or two genomes. (B) The triplet network was split into triplet network modules with specific expression patterns for genome expression bias (Figure S11). (C) The boxplots show subgenome-wise expression strength of triplet members in a *F. graminearum*-responsive triplet module (highlighted in A and B) under the given conditions for NIL1 (Fg: treatment with *F. graminearum*; M: mock treatment; 30: 30hpi; 50: 50 hpi). Within this module, expression in response to the fungus was dominated by the D subgenome, which was also observed for NIL2, NIL3, and NIL4 (Figure S12).

To investigate some of the dynamics in the observed gene expression bias, we inferred a gene coexpression network for triplet genes. Expression bias between the three subgenomes was then captured by a weighted average and nodes were colored according to this average (Figure 6A). The network contained eight modules with distinct expression patterns (Figure 6B and Figure S11). Most of the modules were defined by the expression in one dominant subgenome or by the pair-wise domination of the AB, BD, or respectively the AD subgenomes. One module of the triplet network included triplets with strong transcriptional responses to *F. graminearum* at 30 hpi and 50 hpi (highlighted in Figure 5, A and B). For genes within this module, responses were more pronounced for triplet members in the D subgenome, whereas the A and B subgenome contributed about equally in expression strength (Figure 6C). With respect to the D subgenome dominance, no differences between the four NILs were observed within this module (Figure S12). To investigate whether the more-pronounced reaction of the D genome is also reflected by the greater expression of pathogenesis-related genes, we observed expression differences for genes encoding NB-ARC domains (IPR002182). Although the number of gene family members are dominated by the A and the B subgenome, genes from the D subgenome are significantly greater expressed than homeoalleles from the A subgenome and in many cases also than those from the B subgenome (Figure S13 and Table S11). Similar observations were made for the NBS-LRR genes, although the differences were not as pronounced (Figure S14 and Table S11). Overall, our observations indicate a pronounced role of the D subgenome in response to *F. graminearum*.

## Discussion

### Combined analysis of metabolomics and transcriptomics data

The recent release of the bread wheat reference genome sequence and annotation includes an almost-complete wheat gene set sorted into chromosome-arms (Mayer *et al.* 2014). Both features, the completeness of the resource, and the possibility to assign these genes to genomic regions surpasses by far any previous reference gene sets as a mapping reference for RNA-seq studies. This significantly improved mapping reference, combined with metabolomics data after inoculation with DON (Warth *et al.* 2015) and novel metabolomics data after inoculation with *F. graminearum*, was the main motivation for this study and for revisiting existing data. All of the included experiments used the same *F. graminearum* isolate and bread wheat near-isogenic material (differing in *Fhb1* and *Qfhs.ifa-5A*) and are based on similar protocols for infection and tissue harvesting. Differences existed in the applied amount of spores between the transcriptomics experiment (500 conidia spores/floret) and metabolomics experiment (100 conidia spores/floret). Both concentrations suffice to successfully establish infection. In many biological reactions a stress trigger level must be reached to initiate a process. In complex processes it is often advantageous that all consecutive steps of a response occur automatically and are more or less “programmed.” Applying sufficient conidia to initiate the plant response probably will trigger the whole process. Therefore we do not expect significant differences in the recorded metabolomics and transcriptomics data sets (an example for phenylalanine and is given in Figure S15). Comparable transcriptomics and metabolomics studies have used a wide range of concentrations from 200 to 1000 conidia/floret to elicit a defense response to FHB (Jia *et al.* 2009; Gottwald *et al.* 2012; Foroud *et al.* 2012; Zhuang *et al.* 2013; Schweiger *et al.* 2013; Steiner *et al.* 2009; Gunnaiah *et al.* 2012; Diethelm *et al.* 2012). However, we cannot fully exclude the possibility that the observed metabolite changes, *i.e.*, for amino acids may have been recorded earlier at greater inoculum concentrations. The Bayes score reporting differences in metabolite abundances between treatments considers all time points by comparing differences between models of individual treatments. Possibly, earlier changes resulting from greater concentrations would have influenced the time course models resulting in higher scores indicating even larger differences to the control mock models. However these changes would not alter the reported results here, as difference in score may also reflects the delay in/of metabolite changes but certainly highlights the presence of underlying metabolism.

In a previous transcriptomic study (Schweiger *et al.* 2013) we used very early and late time points for expression analysis (8, 24, and 72 hpi). Although the early time points provided only few differentially expressed genes, at 72 hpi a large and general response was detected for the susceptible lines. Apparently, resistance relevant reactions likely happen before 72 hpi. In the present study we chose two earlier time points, 30 and 50 hpi, to better capture the resistant reaction with two time points that encompass the onset of the production of larger amounts of DON (Pritsch *et al.* 2000, Audenaert *et al.* 2013). *Fhb1* is closely linked to resistance against DON and given the investigated NILs segregating for *Fhb1* these time points are appropriate to investigate related changes. The present metabolomics data describe five time points covering a time span of 96 hr and thus embraces both critical time points captured by RNAseq. Although the individual time points (0, 12, 24, 48, 96 hpi) do not overlap perfectly with 30 and 50 hpi measured in the transcriptomics experiment, they allow modeling a dynamic behavior which can be brought into context with the measured gene expression data. We approached this by avoiding direct comparisons between data sets at specific time points, but compare single time point observations for the RNAseq data with the time course-derived Bayesian score. As such the scores are time-point independent and provide a very reliable means to report changes in relation to treatment.

We analyzed the gathered data on different tiers: I. Given that our metabolomics analysis focused on metabolites derived from the well-annotated primary metabolism, we were able to integrate genotype-specific changes in metabolite abundances with differential transcript abundances of genes involved in the respiratory chain, glutamate metabolism, and amino acid biosynthesis. II. Homeoalleles of hexaploid bread wheat can be identified in the chromosome-sorted IWGSC gene set. We made use of these to investigate differences in the subgenome-wise contributions to defense response. III. Compared with our previous analysis (Kugler *et al.* 2013), the gene coexpression network here includes significantly more genes, as a result of the IWGSC bread wheat reference genome sequence, and allows allocating genes to their corresponding genomes. Therefore, we can provide a more complete and detailed picture on the genome-wide pathogen response and the corresponding dynamics.

In line with our previous observations in Kugler *et al.* (2013) two network modules, representing an early and a late response to the pathogen, were found in the coexpression network. Given the improved gene annotation, we could now further refine these data and provide a more comprehensive functional description of the corresponding genes and the respective pathways, while also taking subgenome contributions into account. For instance, using the current bread wheat annotation, we identified a pathogen-responsive network module (B) enriched for WRKY transcription factors (one-sided Fisher’s test based on the human readable description line; FDR-adjusted *P* < 0.001). In our previous study, such enrichment was also observed for such a generally pathogen responsive network module. Moreover, expanding in comparison with the previous approach, we were able to detect and characterize QTL-specific modules, which were investigated for *F. graminearum*-responsive genes mapping to chromosome-arms harboring the respective QTL.

### Increases in respiration and amino acid biosynthesis as a response to *F. graminearum* and DON

The plant defense against pathogens requires the *de novo* synthesis of a plethora of secondary metabolites and defense-related proteins as well as the fortification of cell walls and antagonizing the effects of oxidative stress (Walter *et al.* 2010). As such, it is energy intensive and requires elevated rates in respiration (Bolton 2009). Mounting a successful defense response may rely on the efficiency of these mechanisms to supply the required substrates, but alterations in the primary metabolism have also been suggested to contribute to defense by themselves (Schwachtje and Baldwin 2008). For instance, glucose and hexokinase activity induce PR genes in *Arabidopsis thaliana* (Xiao *et al.* 2000), whereas silencing of the hexokinase 1 in *Nicotiana benthamiana* led to increased levels of H_2_O_2_ and programmed cell death−associated transcripts (Kim *et al.* 2006). Also, much evidence has been gathered for similar mechanisms involving the metabolism of several amino acids as well as lipids and the photorespiratory chain (Rojas *et al.* 2014). Our analysis aimed to reconstruct such activities in the bread wheat respiratory chain, in glutamate metabolism and in the biosynthesis of amino acids.

We found glycolysis, the pentosephosphate pathway, and the TCA cycle strongly induced in response to the pathogen, which might be fueled by the invertase-mediated cleavage of sucrose into glucose and fructose. The greater turnover rates in these pathways were visible in increased transcript abundances despite the—in many cases—unchanged pathway intermediate metabolites. In this respect, the response to *F. graminearum* is similar to the general model of the plant primary metabolism under pathogen attack (Bolton 2009). Upon carbon starvation, plants may procure additional carbon from abundant amino acids for respiration (Araújo *et al.* 2011), but this seems not to be the case in the wheat/*F. graminearum* interaction. Warth *et al.* (2015) have reported increased amino acid abundances in response to DON, hypothesizing that this may either be due to the increased biosynthesis of amino acids or that amino acids stem from degradation of unfinished peptide chains as a consequence of the ribotoxic effect of DON. In the gene expression data, we found strong indications for the increased biosynthesis of amino acids, likely reflecting the efforts of keeping up the protein biosynthesis to counteract the effect of DON.

Key biosynthesis genes for all amino acids except proline, lysine, serine, and asparagine were more abundant in response to the pathogen. Although we did not find evidence for the increased biosynthesis of serine or proline in the transcriptomic data, DON-inoculated samples showed strong differences in the abundance of serine and proline compared with mock treatment as indicated by the Bayes score (highlighted in red in Figure 4). Levels of both amino acids are strongly increased after treatment with DON (Warth *et al.* 2015). In particular, proline was reported to be more abundant under different stresses (Sharma and Dietz 2006). Proline accumulated in tissues surrounding hypersensitive lesions caused by *Pseudomonas syringae* on *Arabidopsis thaliana*, leading to the assumption that it may play a role in quenching free radicals (Fabro *et al.* 2004). It also holds a role in nitrogen transport in the phloem, as its levels are possibly linked to GS1 activity (Brugière *et al.* 1999). Other proteinogenic amino acids such as the phenolic amino acids and amino acids derived from aspartate were also highly changed in response to DON. Yet, aspartate itself, as well as the functionally linked pools of asparagine, glutamine and glutamate, were largely unchanged. Glutamate pools remain largely unchanged under stress conditions (Forde and Lea 2007), and we speculate that this could also be true for the closely linked aspartate. Glutamine and asparagine levels in contrast can be subject to change due to active influx of these compounds as nitrogen-sources into the sink tissue (Masclaux-Daubresse *et al.* 2006). Other amino acids with less pronounced changes such as serine or alanine may in fact be remobilized into the respiratory chain as suggested by (Araújo *et al.* 2011). Concerning the suggested two alternative models for increased amino acid abundances (Warth *et al.* 2015) our combination of transcriptomic and metabolomic data provided clear evidence that a higher tRNA-ligase activity is supported by increased amino acid biosynthesis - yet not all amino acids biosynthesis pathways are similarly affected.

### Increased activity in the glutamate metabolism: a possible role for *Qfhs.ifa-5A*−mediated resistance?

Our previous study (Kugler *et al.* 2013) found the activity of glutamate regulated Ca^2+^ channels associated with *Qfhs.ifa-5a*. Now using the almost complete bread wheat genes, we were able to fully reconstruct changes in the glutamate metabolism during defense. The *F. graminearum*-responsive GS1 and NADPH-dependent GOGAT genes are not part in metabolizing newly synthesized ammonium from photosynthesis but facilitate the transport of nitrogen in the form of glutamine through the phloem into sink/infected tissue (Masclaux-Daubresse *et al.* 2010). Seifi *et al.* (2013) suggested two different roles in pathogen defense for the metabolism of glutamate, which may either act toward depleting infected tissue from nitrogen compounds to prevent these from being scavenged by the pathogen or it may assist the cell to endure the disease by hauling energy equivalents into the infected tissue. The latter is characterized by the increased activity of the GOGAT/GS cycle, the GABA shunt, and glutamate deyhdrogenase genes, whereas aspartate transaminases and asparagine synthases are less active. Our data suggest the bread wheat defense against *F. graminearum* aims to endure the disease by strengthening the TCA cycle and supplying carbon/nitrogen for the biosynthesis of secondary metabolites: GS1-generated glutamine enters the infected tissue as an additional carbon and nitrogen source. In sink tissue glutamine is metabolized to glutamate by NADH-dependent GOGAT and then further decomposed into 2-oxoglutarate by the also highly *F. graminearum*-responsive glutamate dehydrogenases (GDHs). Although GDH can perform the reverse reaction to additionally assimilate ammonium under given conditions, the more likely reaction is the deamination of glutamate into 2-oxoglutarate as an anaplerotic reaction to fuel the TCA cycle (Masclaux-Daubresse *et al.* 2006). Abundant ammonium from GDH activity may be reused by GS1 located in the infected tissue.

Several of these described genes showed differential expression patterns for lines differing in *Qfhs.ifa-5A*. Transcript abundances for NADPH-GOGAT and GDH genes were greater in *F. graminearum*−treated samples at 30 hpi for lines harboring *Qfhs.ifa-5A*. Potentially, these lines react earlier to the influx of glutamine and provide earlier an increased amount of 2-oxoglutatare to the TCA cycle (II, III, and V in Figure 4). Such differences also were observed for several TCA cycle genes (aconitases, citrate synthases, succinate dehydrogenases) as well as malic enzymes, which provide additional pyruvate for the TCA cycle. Similarly, associated metabolite levels are changed in response to DON and the fungus only for lines lacking this earlier reaction, which we observed for the *Qfhs.ifa-5A*-lines (Figure 4). TCA intermediate substances are subject to high turnover rates and concentrations tend to be stable (Sweetlove *et al.* 2010). The required increased flux in response to the pathogen seems to be more efficiently met by the earlier action of *Qfhs.ifa-5A*-lines. In contrast, the adaptation to *F. graminearum* in non-*Qfhs.ifa-5A* lines leads to the observed changes in pool levels. However, the large variances in the measurements of metabolites do not allow a further interpretation of the present data. Not all genes changed for *Qfhs.ifa-5A* act earlier in response to the pathogen: Pyruvate dehydrogenases and malate dehydrogenases were less strongly changed at 50 hpi in response to *F. graminearum* in *Qfhs.ifa-5A* containing lines (Figure 4). How these expression patterns fit into the proposed mechanism remains unclear. For a more detailed interpretation of these observations more comprehensive, preferably longitudinal expression profiles will be needed.

The observed changes for the TCA cycle and glutamate metabolism could contribute to a greater “endurance” in *Qfhs.ifa-5A* lines and thus be part the resistance mechanism encoded by the QTL. Among the genes constitutively changed for the QTL encoded on 5A in module C two amino acid permeases (AAP, isoforms 6, *Traes_5AS_776E1FEE4* and 8, *Traes_5AS_073CAB1CC*) could contribute to the greater influx of amino acids from the phloem and provide substrates for the observed QTL-associated changes. In *A. thaliana* AtAAP6 regulates the phloem amino acid composition and AtAAP8 is involved in seed development (Tegeder and Rentsch 2010). AtAAP6 was reported to be expressed in sink tissue with a high affinity for neutral amino acids and other amino acids with acidic side chains (Hunt *et al.* 2010). Also AtAAP8 has a high affinity to aspartate and glutamate (Schmidt *et al.* 2007). Because of the potentially large pericentromeric introgression harboring the QTL many other genes that show constitutive expression differences cannot be ruled out as putative candidates. This list (Table S10) also includes candidates from our previous studies (Schweiger *et al.* 2013, Kugler *et al.* 2013) including a lipid transfer protein, which shows among the highest expression differences.

### Narrowing down single-gene candidates is aggravated by the susceptible Chinese Spring mapping reference

We have made use of a combination of coexpression patterns, differential expression analysis, and chromosome location to narrow down the list of candidates for *Fhb1*. Most genes specifically expressed for *Fhb1* exhibited a constitutive expression pattern (module D). Although many of these genes map to chromosome 3B a closer inspection showed that none of the modules hub genes mapped within close vicinity of the genomic region carrying the susceptible *Fhb1* locus of cultivar Chinese Spring (contig ctg0954 [GenBank:FN564434] (Choulet *et al.* 2010). It is unclear how large the introgressed region carrying *Fhb1* is. Possibly several of the reported genes are distant from the locus and QTL unrelated: Mapping against the complete 3B chromosome sequence (Choulet *et al.* 2014) placed *Traes_3B_3A70D33A6*, a receptor-like protein kinase, at position 10.095.372 bp and *Traes_3B_6A585354F*, a protein kinase, at position 9.817.008 bp, which is about 17 Mbp distal to the *Fhb1* marker Umn10 (27.605.772 bp). Several other genes in module D mapped to ctg0954, but only three within the QTL confidence interval between flanking markers *Sts32* and *Sts189*. All three are only weakly expressed and corresponding transcripts are more abundant in lines harboring the susceptible QTL allele (*Traes_3B_07980E2CE*, *Traes_3B_0D8C9A632*, *Traes_3B_CDF3C9680*). Similarly, candidates from module E, which groups *Fhb1*-specific and *F. graminearum*−responsive genes (*Traes_3B_5088D482E*, 19.748.084 bp, SINA-like 11 E3 ubiquitin-protein ligase and *Traes_3B_6E28B451A*, 4.483.234 bp, unknown) are too distant from the marker to be considered candidate genes based on the Chinese Spring reference.

The absence of closely mapped genes does not necessarily mean that the elusive *Fhb1* gene remains unrecognized by the IWGSC wheat gene set, which is also based on the susceptible Chinese Spring cultivar. It may be that the resistance gene is only present in the resistant genotype and would not map onto the contig. Likely reads derived from genes not represented in the gene set will map to close homologs or in case of hexaploid wheat to the next homoelog on sister chromosomes should they exist. For example *Traes_3DS_2BD8DC857* specifically expressed for *Fhb1* containing lines maps to the homoelogous region of *Fhb1* on chromosome 3D. Possibly, the susceptible reference genotype Chinese Spring lacks such a gene in the *Fhb1* region and 3B-specific reads map to this putative homeolog. The possible absence of this gene in the susceptible cultivar Chinese Spring may derive from pseudogenization or small chromosomal rearrangements (Bennetzen and Ramakrishna 2002), which may occur even between different varieties of the same species (Feuillet and Salse 2009). Liu *et al.* have compared the synteny of the genetically mapped locus of *Fhb1* to the rice and barley physical maps and did find evidence for rearrangements based on marker collinearity in this region (Liu *et al.* 2006). Possibly, the gene content and/or gene order of the genomic region containing the resistant *Fhb1* locus does not follow the established Chinese Spring reference cultivar. This would also allow to hypothesize that candidate genes that mapped in this study distant to the *Fhb1* marker Umn10 in Chinese Spring could in fact be located within the QTL confidence interval in *Fhb1*-containing genotypes.

On the other hand, this study has considered high-confidence gene models only. Low confidence genes in wheat comprise a large number, more than 100,000, of putatively fragmented gene models, pseudogenes, and repeat associated elements for which little evidence for functionally expressed gene products exist. In Schweiger *et al.* (2013) we have measured gene expression using the Affymetrix wheat GeneChip, yielding four differentially expressed transcripts mapping to the region of *Fhb1*. Two of these genes corresponded to low confidence genes (Ta.6066.2.S1_a_at: *Ta3bMIPSv2Loc009233*; Ta.22694.1.A1_at: *Ta3bMIPSv2Loc008006*) and only one, Ta.28185.1.S1_at, had homology to a high confidence gene (*Traes_3B_05EEE7D3F1*). This is also the only gene mapping to the QTL confidence interval between markers *sts32* and *sts189*. All genes are expressed in a constitutive manner for the absence/presence of the introgressed QTL region, which matches the reported findings. *Traes_3B_05EEE7D3F1* is higher expressed for the susceptible QTL allele. One of the reported differentially expressed probe sets (TaAffx.12498.2.S1_at) was not included in either data set. Possibly these low confidence genes are relevant for the QTL activity and should be considered once the genomic region of a *Fhb1*-containing cultivar is resolved and available.

Genetic approaches to map these genes in materials segregating for *Fhb1* are required to narrow down this list of candidates. Zhuang *et al.* (2013) have mapped the expression traits of one out of 47 FHB investigated resistance candidate genes in an expression QTL study to the *Fhb1* locus (10.1094/MPMI-10-12-0235-R). However, a BLAST survey of this putative resistance gene designated *WFhb1_c1* showed that the IWGSC gene set does not include a homolog on chromosome 3B and also their mapped *Fhb1* interval spanning more than 16 cM is large.

### Imbalances in subgenome expression contribution

Polyploidization events present a form of “genomic shock,” which leads to increased transposable element activity and epigenetic silencing (Wendel 2000). Such effects may also be reflected in the expression patterns and the interplay between the A, B, and D subgenomes. An imbalance in the number of disease-resistance genes has been described for tetraploid and hexaploid wheat, with the highest number of genes stemming from the B subgenome (Feldman *et al.* 2012; Fahima *et al.* 2006; Peng *et al.* 2003). Based on the IWGSC annotation, most members of two defense-related gene families were encoded on the A and B subgenomes (Mayer *et al.* 2014). This distribution does not correspond to findings in our data, where in terms of gene expression contributions to the defense response from the D and the B subgenomes dominated over contributions from the A subgenome. Such expression observations might be affected by differences in total gene numbers on the subgenomes. To address this we eliminated this bias by considering only the 1:1:1 homoelogous triplet genes in the triplet gene coexpression network, which showed that genes from the D subgenome are more abundant in response to the pathogen than their A and B counterparts. From these observations and the hub gene-specific subgenome distribution in modules A and B we reason that subgenome D contributions may play a decisive role in overall resistance to *F. graminearum*. This hypothesis could relate to the overall high susceptibility to FHB of tetraploid durum wheat (*Triticum turgidum ssp. durum*, 2n = 4x = 28, AABB). Only single lines have been described which harbor intermediate levels resistance to FHB (Huhn *et al.* 2012; Prat *et al.* 2014). Because durum and bread wheat share the same ancestral A and B subgenomes, the added resistance in bread wheat may stem from D subgenome contributions. Although, most of the relevant resistance QTL in *T. aestivum* have been mapped to the A or B subgenome (Buerstmayr *et al.* 2009), resistances encoded on the D subgenome may well play decisive roles: The D subgenome is much less polymorphic due to its evolutionary only recent addition to wheat and thus resistance contributors may not be segregating in mapping populations. An indication for its relevance comes from *Aegilops tauschii*, the contributor of the D subgenome to wheat, which has been used widely in the generation of synthetic hexaploid wheats from crosses with tetraploid species such as *Triticum turgidum*. The addition of the D subgenome has improved resistances against a variety of biotic and abiotic stresses including resistance against FHB in comparison to the tetraploid parental line (Mujeeb-Kazi *et al.* 2008). Whether these resistance genes are in effect in *T. aestivum* remains to be shown. However, we also observed a slight bias toward the A genome for triplets, where only a single copy was responsive to the pathogen. Overall, it appears that the response to the pathogen is distributed between the different subgenomes, and more data is needed to generalize findings of subgenome bias in the context of bread wheat pathogen response.

## Supplementary Material

Supporting Information
