# Supplementary material for: Joint Transcriptomic and Metabolomic Analyses Reveal Changes in the Primary Metabolism and Imbalances in the Subgenome Orchestration in the Bread Wheat Molecular Response to Fusarium graminearum
Source: G3 (Bethesda). 2015 Oct 4;5(12):2579–92. doi: 10.1534/g3.115.021550 (PMC4683631; doi:10.1534/g3.115.021550)
Supplement: Supporting Information [file supp_5_12_2579__index.html]

Joint Transcriptomic and Metabolomic Analyses Reveal Changes in the Primary Metabolism and Imbalances in the Subgenome Orchestration in the Bread Wheat Molecular Response to Fusarium graminearum — Supporting Information 

# Joint Transcriptomic and Metabolomic Analyses Reveal Changes in the Primary Metabolism and Imbalances in the Subgenome Orchestration in the Bread Wheat Molecular Response to *Fusarium graminearum*

## Supporting Information for Nussbaumer *et al.*, 2015

**Files in this Data Supplement:**

- Supporting Information - Figures S1-S15 and Tables S1-S11 (PDF 1.2 MB)
- Figure S1 - Picking of a soft-thresholding power beta and analysis of scale-free topology (PDF 239 KB)
- Figure S3 - Enrichment for differentially expressed genes within the co-expression modules (PDF 340 KB)
- Figure S4 - Scoring of the differences in treatment for the comparison between DON and water treatment on the metabolomics data (PDF 352 KB)
- Figure S5 - Scoring of the differences in treatment for the comparison between *Fusarium graminearum* and water treatment on the metabolomics data (PDF 252 KB)
- Figure S6 - Expression of Gluthamine synthetase genes (PDF 211 KB)
- Figure S2 - Picking of a soft-thresholding power beta and analysis of scale-free topology for the triplet network (PDF 239 KB)
- Figure S15 - A: Time course relative abundances for phenylalanine. Left panel corresponds to mock treated samples, right corresponds to Fusarium graminearum treated samples., C1-C4 = NIL1-NIL4. B: Eigengene representation of the phenylalanine biosynthesis gene encoding prephenate dehydratase. C1-C4 = NIL1-4. M= mock, F= Fusarium, 30, 50 = 30 and 50 hai (PDF 228 KB)
- Figure S7 - Visualization of the chromosomal positioning of the modules D (royalblue) and C (darkgreen) and the corresponding chromosome-arm enrichment with chromoWIZ (http://pgsb.helmholtz-muenchen.de/plant/chromoWIZ/) (PDF 223 KB)
- Figure S8 - Constitutive expression of hub genes on 3B and 3D (PDF 331 KB)
- Figure S9 - Subgenome-wise contribution to differentially expressed genes (PDF 393 KB)
- Figure S10 - Analysis of triplet expression (PDF 196 KB)
- Figure S11 - Module-wise expression in the triplet network (PDF 225 KB)
- File S12 - Condition-wise expression patterns for each of the four genotypes in the 'green' triplet network module (PDF 383 KB)
- Figure S13 - Expression of NB-ARC domain containing genes (PDF 305 KB)
- Figure S14 - Expression of NBS-LRR genes (PDF 308 KB)
- Table S1 - List of identified metabolites (.xlsx, 57 KB)
- Table S2 - RNA-sequencing mapping statistics (.xlsx, 53 KB)
- Table S3 - List of differentially expressed genes in response to F graminearum treatment (.xlsx, 2.3 MB)
- Table S4 - List of differentially expressed genes comparing NILs against NIL4 (.xlsx, 158 KB)
- Table S5 - Gene family and pathway mapping (.xlsx, 80 KB)
- Table S6 - Metablite measurements (.xlsx, 204 KB)
- Table S7 - Scoring for differences in treatment (.xlsx, 71 KB)
- Table S8 - WGCNA network module Gene Ontology enrichment results (.xlsx, 130 KB)
- Table S9 - Gene expression per condition for selected gene families (as FPKM) (.xlsx, 168 KB)
- Table S10 - List of 5A candidate genes from WGCNA module C "darkgreen" (.xlsx, 55 KB)
- Table S11 - Differences in subgenome-specific characteristics for NBARC and NBS-LRR gene families (.xlsx, 13 KB)
