# Supplementary material for: Joint Transcriptomic and Metabolomic Analyses Reveal Changes in the Primary Metabolism and Imbalances in the Subgenome Orchestration in the Bread Wheat Molecular Response to Fusarium graminearum
Source: G3 (Bethesda). 2015 Oct 4;5(12):2579–92. doi: 10.1534/g3.115.021550 (PMC4683631; doi:10.1534/g3.115.021550)
Supplement: Supporting Information [file supp_g3.115.021550_021550SI.pdf]

Joint transcriptomic and metabolomic analyses reveal changes in the primary metabolism and imbalances in the subgenome orchestration in the bread wheat molecular response to *Fusarium graminearum*

Thomas Nussbaumer[1*], Benedikt Warth[2†], Sapna Sharma[*], Christian Ametz[§], Christoph Bueschl[†], Alexandra Parich[†], Matthias Pfeifer[*], Gerald Siegwart[§], Barbara Steiner[§], Marc Lemmens[§], Rainer Schuhmacher[†], Hermann Buerstmayr[§], Klaus F.X. Mayer[*], Karl G. Kugler[*], Wolfgang Schweiger[§3]

[*]Plant Genome and Systems Biology, Helmholtz Zentrum München, Neuherberg, D-85764, Germany

[§]Institute for Biotechnology in Plant Production (IFA-Tulln), BOKU - University of Natural Resources and Life Sciences, Tulln, A-3430, Austria

[†]Center for Analytical Chemistry (IFA-Tulln), BOKU - University of Natural Resources and Life Sciences, Tulln, A- 3430, Austria

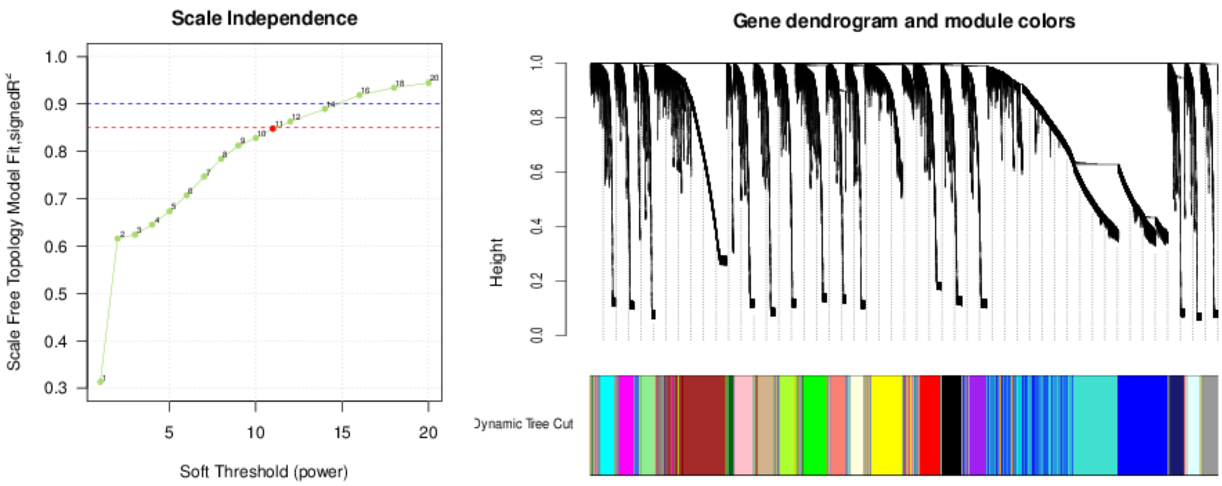

Supplemental Figure 1- Picking of a soft-thresholding power beta and analysis of scale-free topology. Clustering based on the Topological Overlap Matrix.

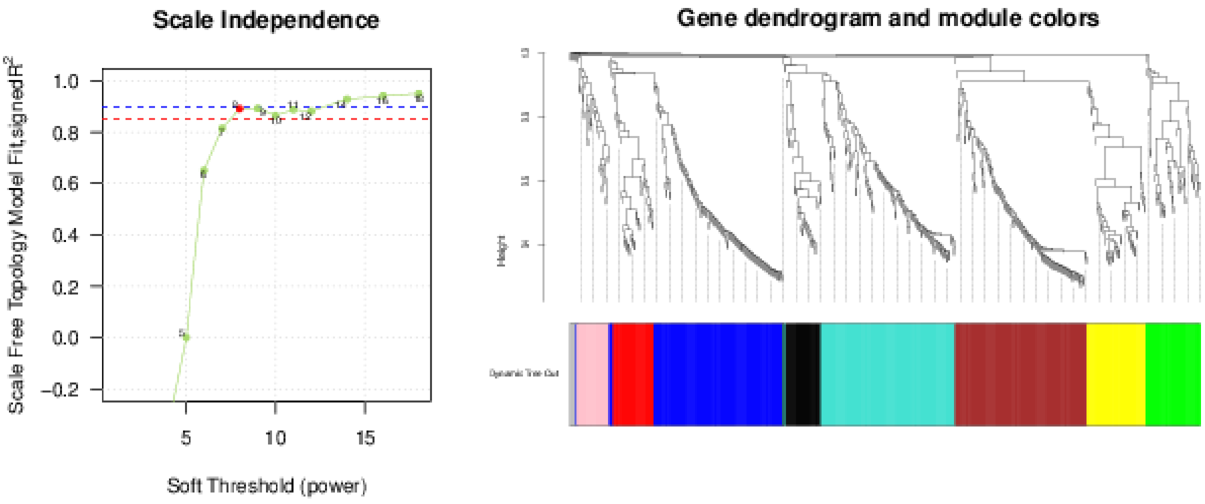

Supplemental Figure 2 - Picking of a soft-thresholding power beta and analysis of scale-free topology for the triplet network. Clustering based on the Topological Overlap Matrix.

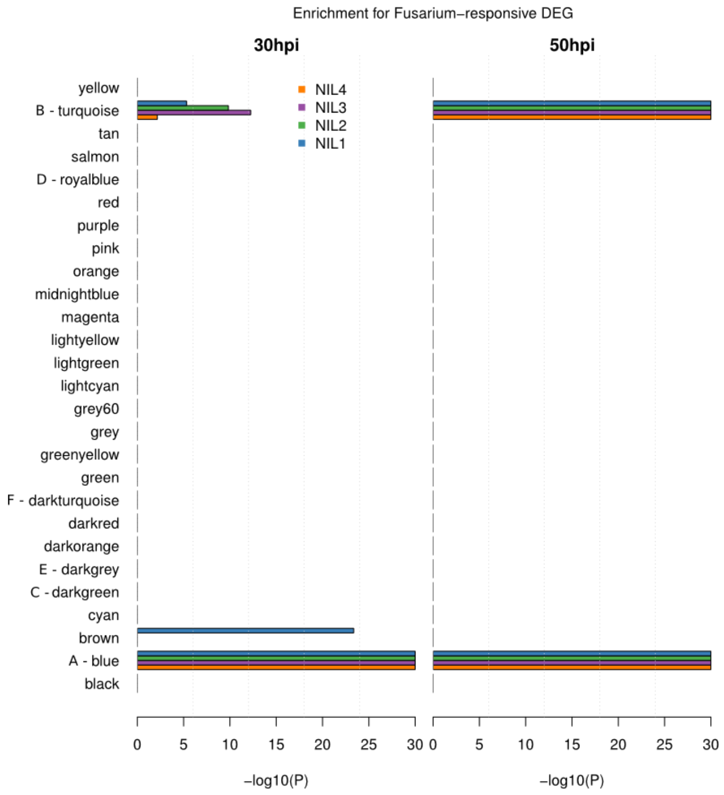

Supplemental Figure 3 - Enrichment for differentially expressed genes within the co-expression modules. FDR adjusted P values from a one-sided Fisher's exact test. For readability the bars were truncated at 10-30. Color names refer to the original WGCNA module labeling.

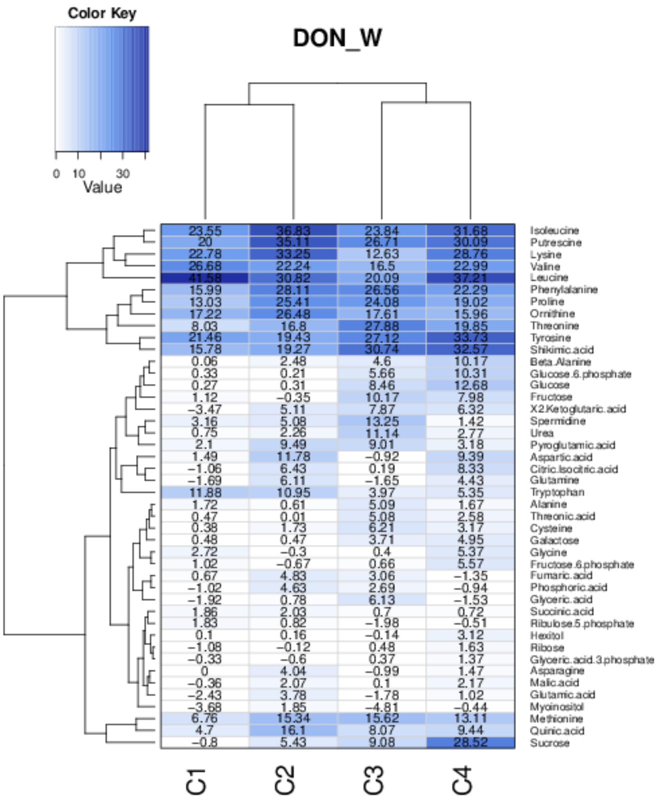

Supplemental Figure 4 - Scoring of the differences in treatment for the comparison between DON and water treatment on the metabolomics data.

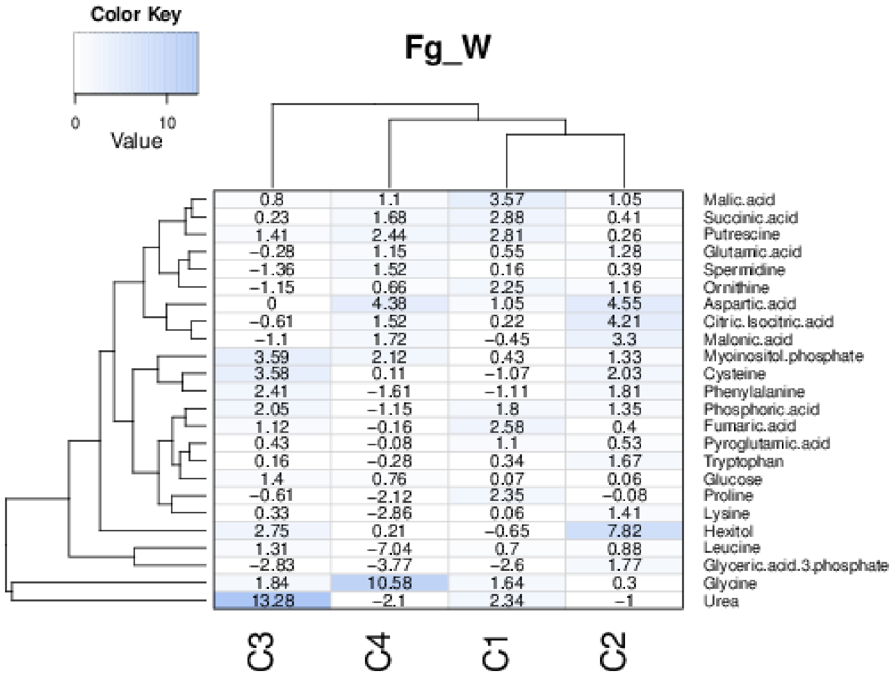

Supplemental Figure 5 - Scoring of the differences in treatment for the comparison between *Fusarium graminearum* and water treatment on the metabolomics data.

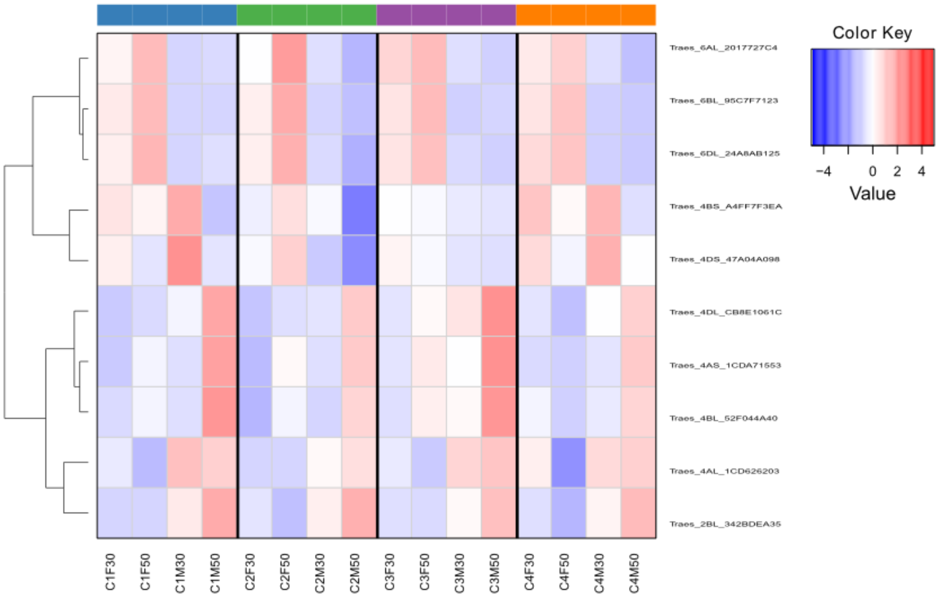

Supplemental Figure 6 - Expression of Gluthamine synthetase genes. Genes were derived by homology to A thaliana genes.

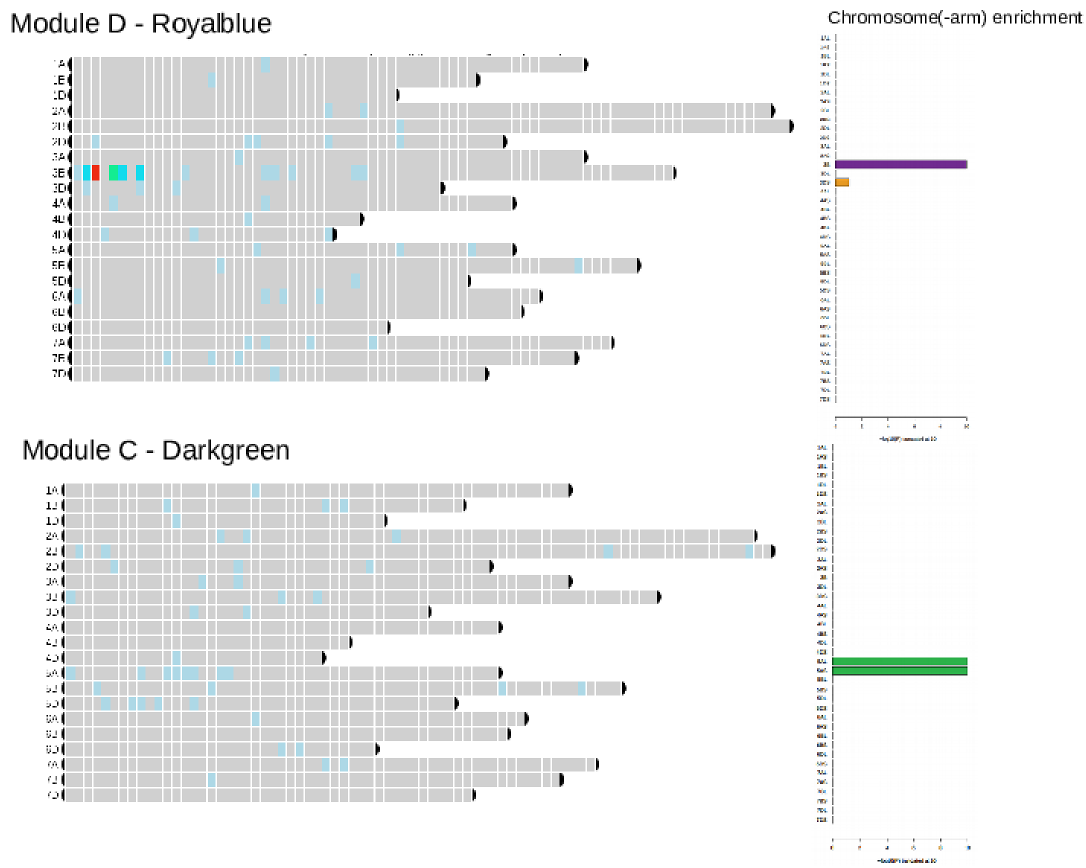

Supplemental Figure 7 - Visualization of the chromosomal positioning of the modules D (royalblue) and C (darkgreen) and the corresponding chromosome-arm enrichment with chromoWIZ (http://pgsb.helmholtz-muenchen.de/plant/chromoWIZ/). Color names refer to original WGCNA module names.

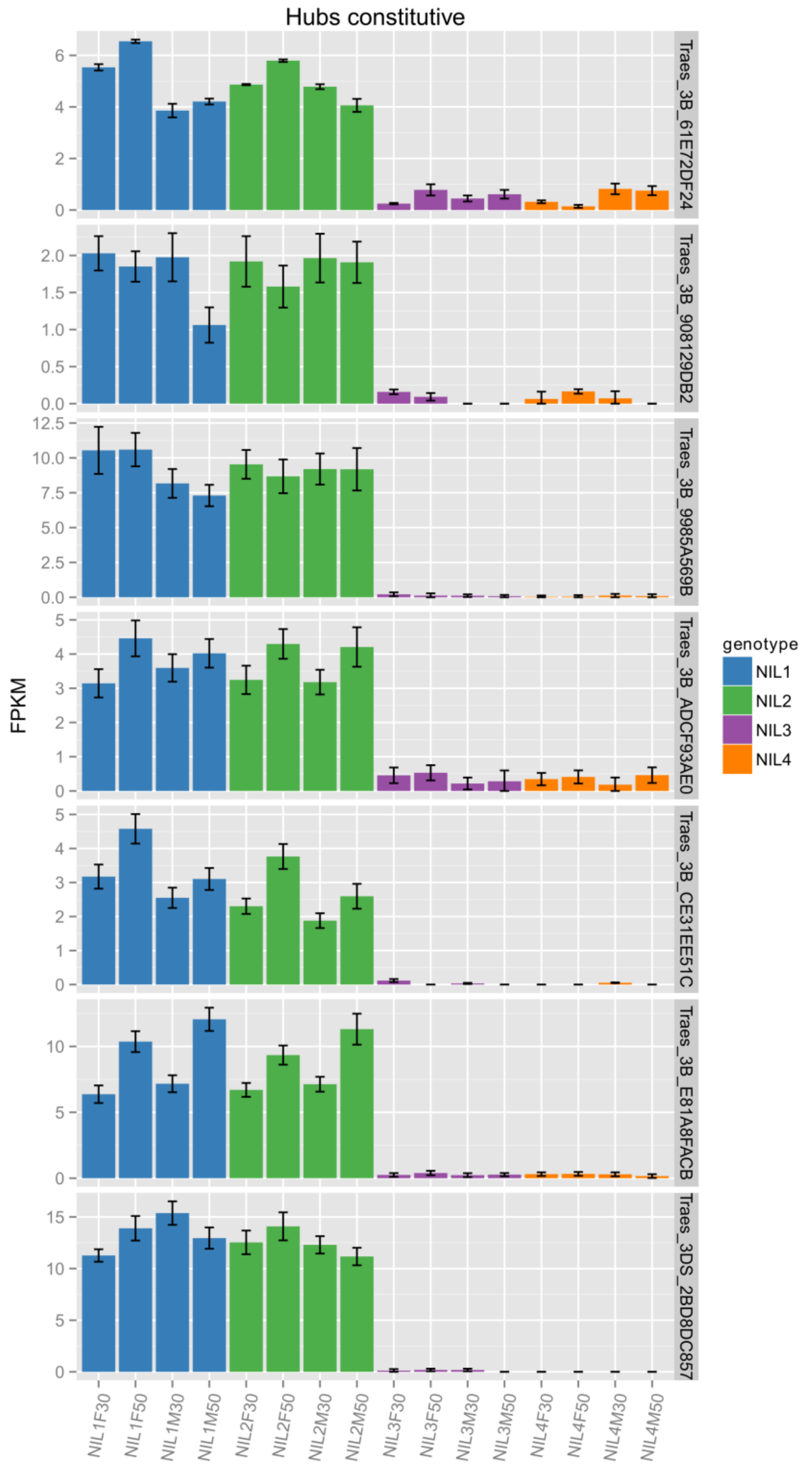

Supplemental Figure 8 - Constitutive expression of hub genes on 3B and 3D.

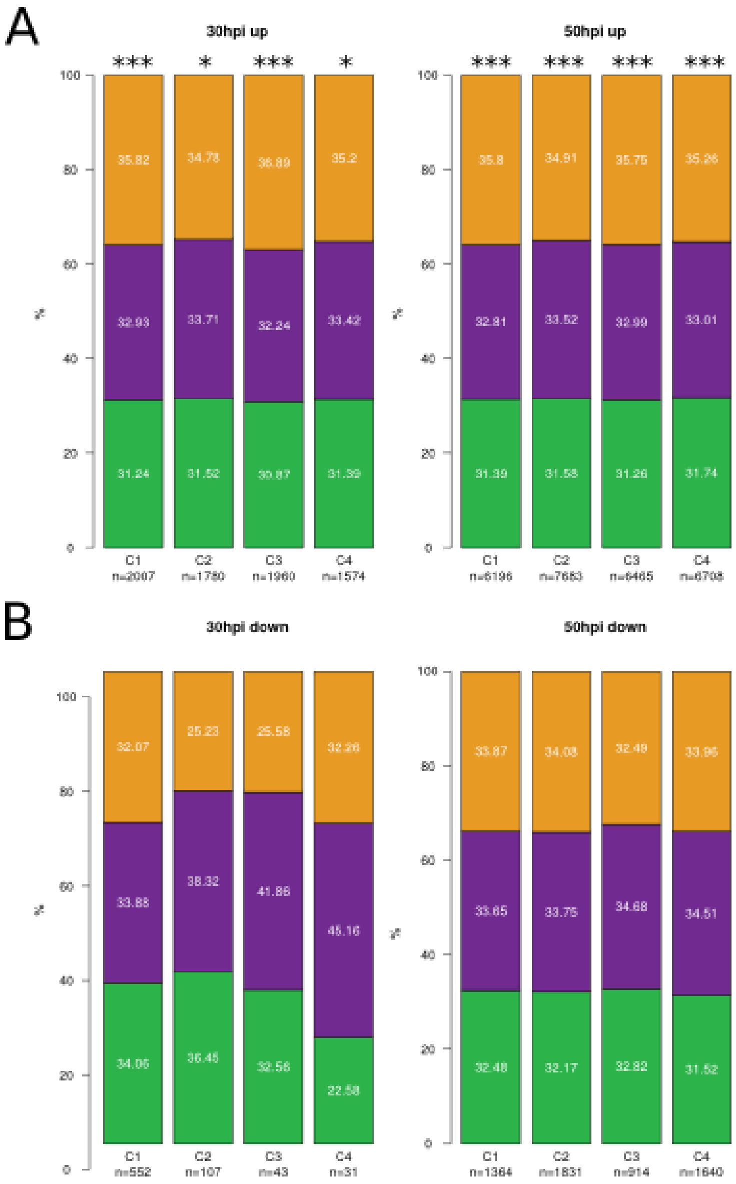

Supplemental Figure 9 - Subgenome-wise contribution to differentially expressed genes. (A) Genes that show increased expression after *Fusarium graminearum* treatment. (B) Genes that show reduced expression after *Fusarium graminearum* treatment.Significance of deviations from the expected distributions was quantified by a chi-squared test against 10,000 random multinomial distributions following the expected A, B, D subgenome distribution from the bread wheat high confidence gene set (* FDR adjusted P < 0.05; *** FDR adjusted P < 0.001). The number of genes with significant changes in expression levels is given as 'n'.

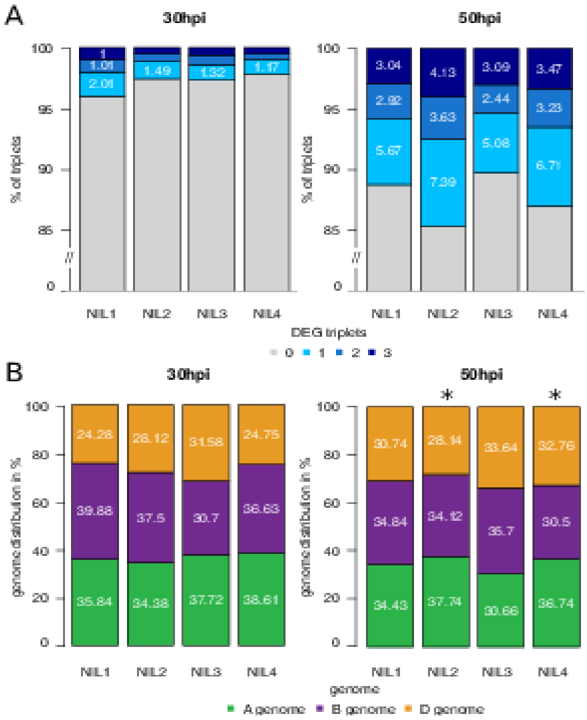

Supplemental Figure 10 - Analysis of triplet expression. (A) Ratio of differential expressed triplet members. (B) Subgenome distribution of triplets with only one members showing differential expression after treatment with *Fusarium graminearum*. Significance of deviations from the expected distributions was quantified by a chi-squared test against 10,000 random multinomial distributions following the expected A, B, D subgenome distribution from the bread wheat high confidence gene set (* FDR adjusted P < 0.05).

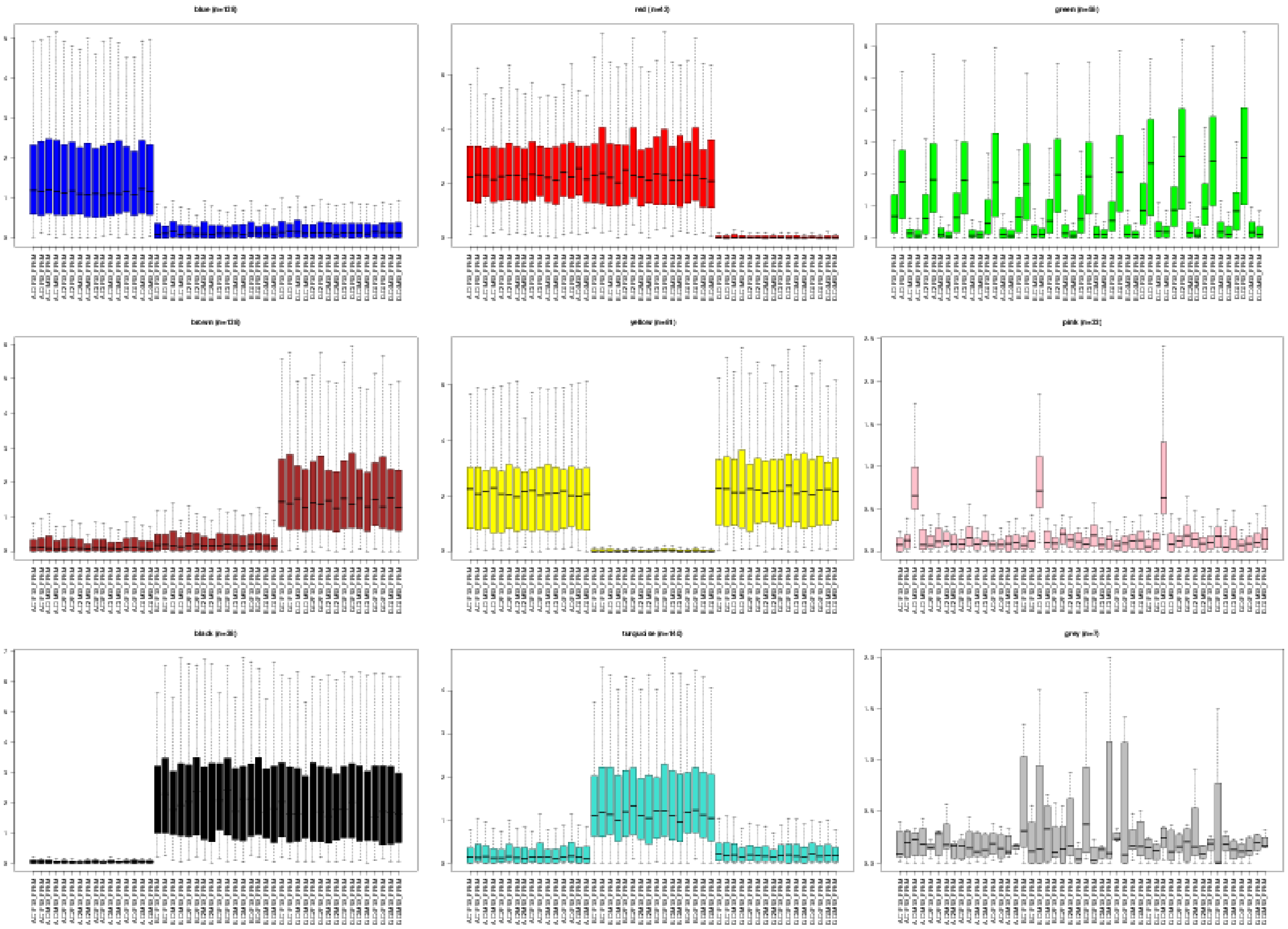

Supplemental Figure 11 - Module-wise expression in the triplet network. The first letter in the names on the x-axis indicates the subgenome (A, B, or D).

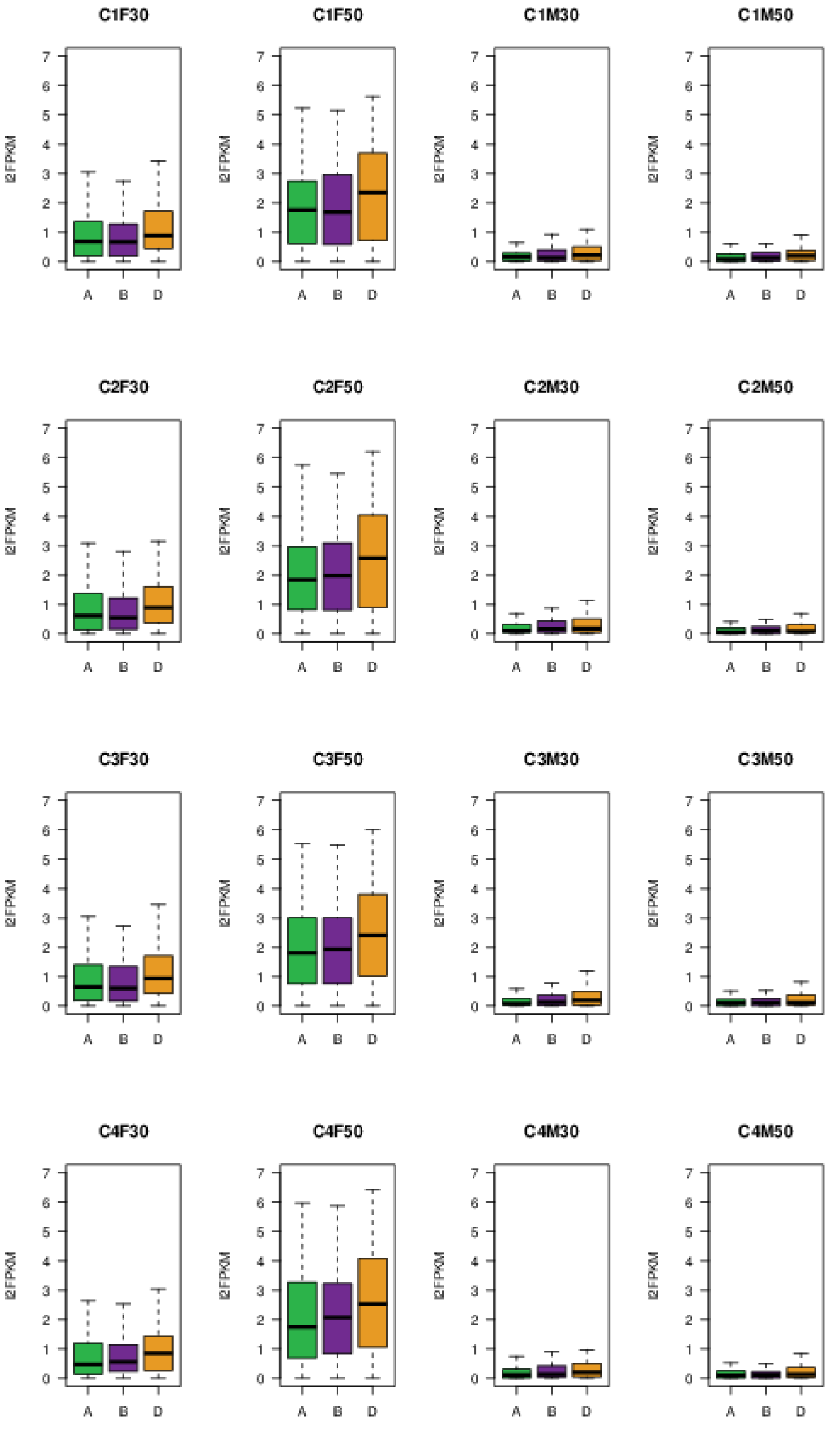

Supplemental Figure 12 – Condition-wise expression patterns for each of the four genotypes in the 'green' triplet network module. (F: *Fusarium graminearum* treatment; M: mock treatment; 30: 30hpi; 50: 50hpi)

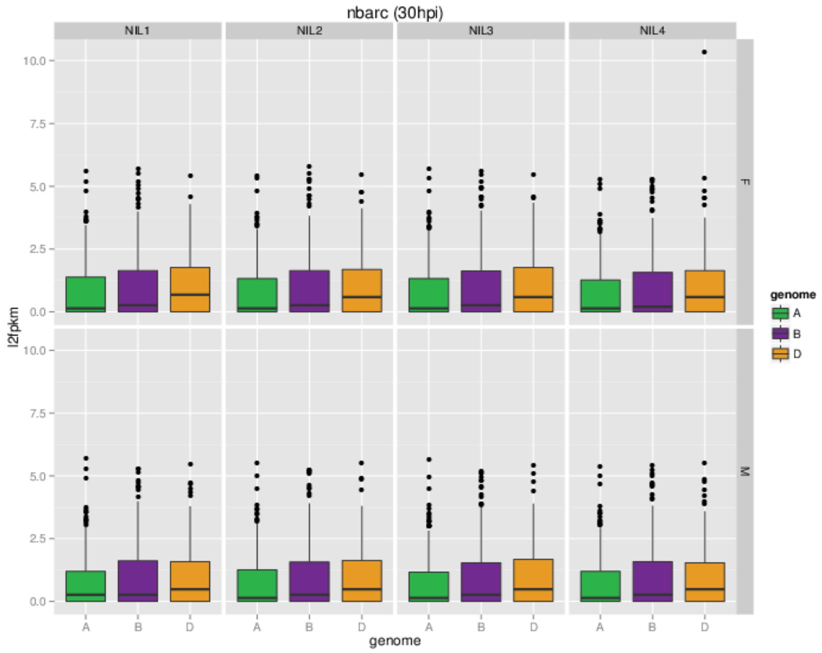

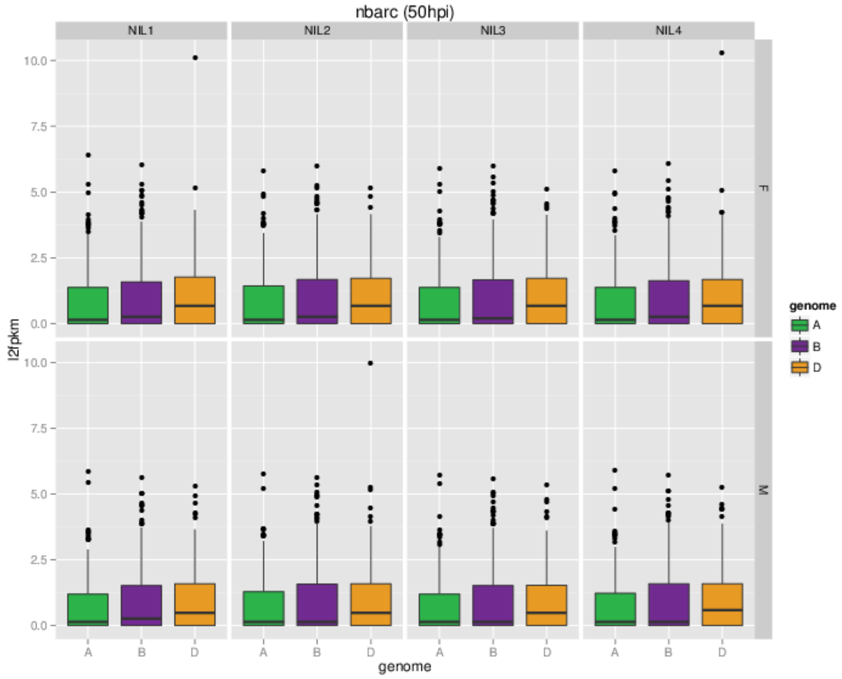

Supplemental Figure 13 – Expression of NB-ARC domain containing genes. Genes were extracted based on the NB-ARC Interpro domain (IPR002182; http://www.ebi.ac.uk/interpro/entry/IPR002182).

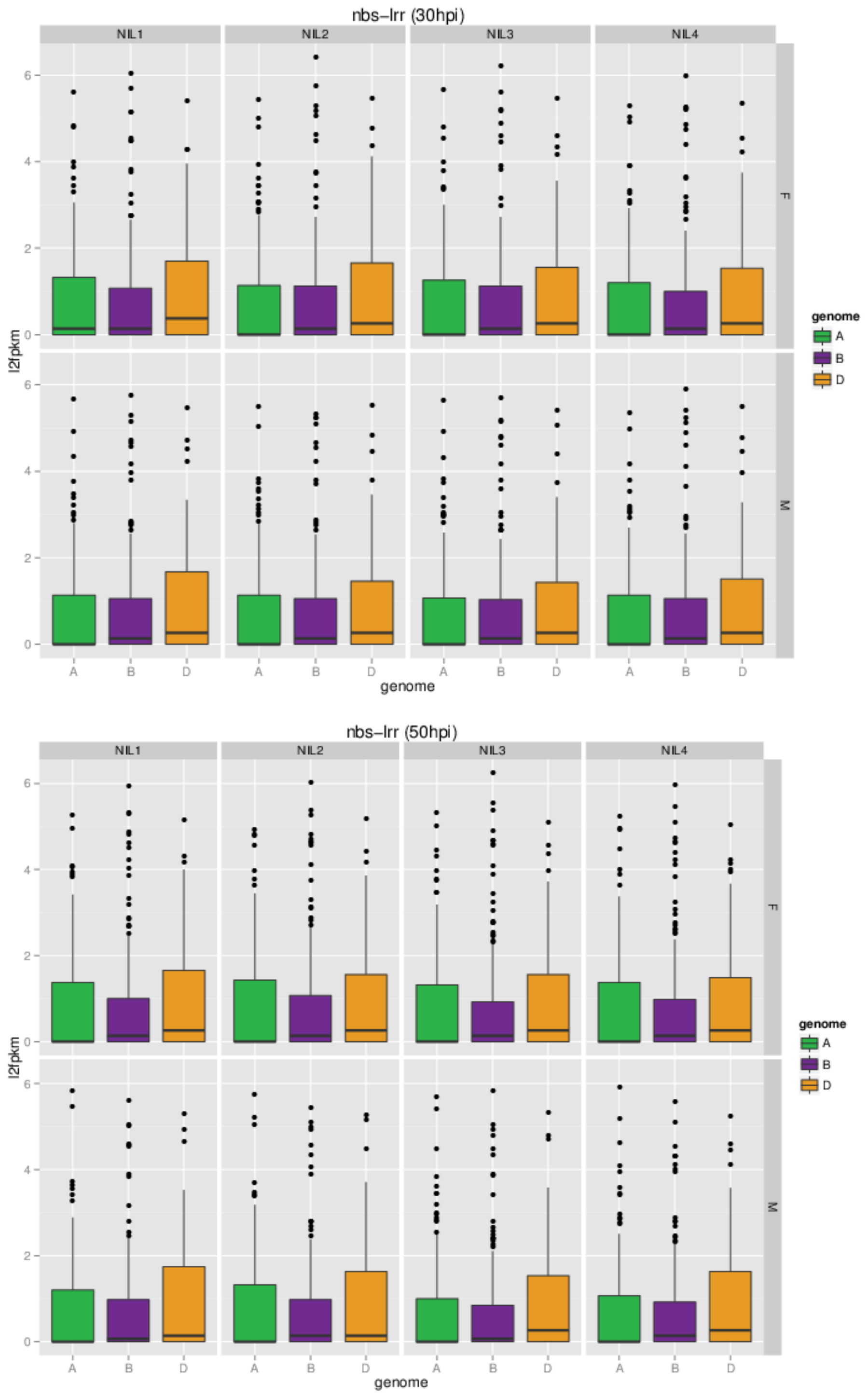

Supplemental Figure 14 - Expression of NBS-LRR genes. Genes were extracted by searching for the term “NBS-LRR” in the IWGSC bread wheat genome annotation AHRD.

A

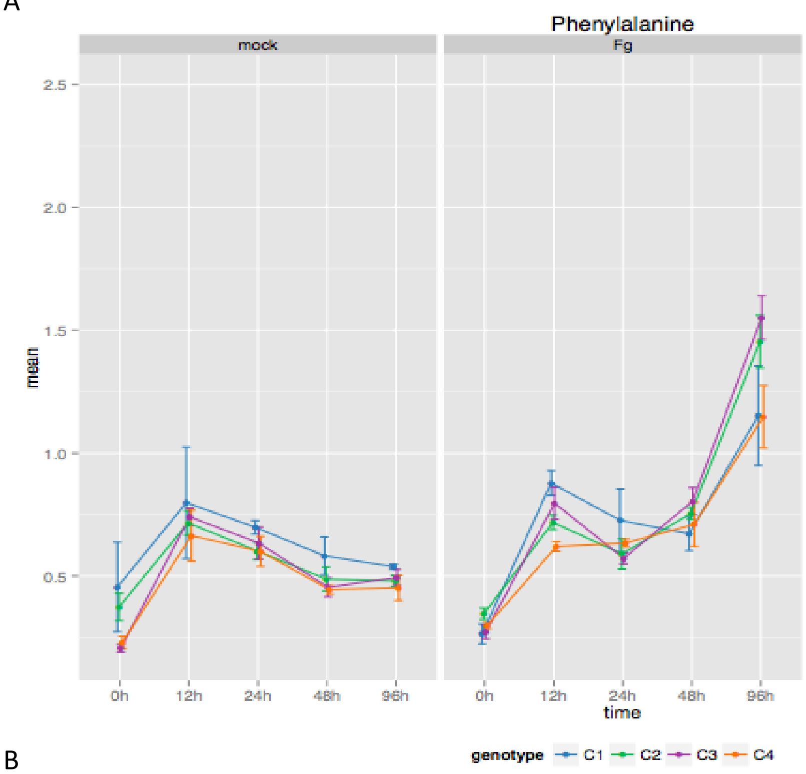

B

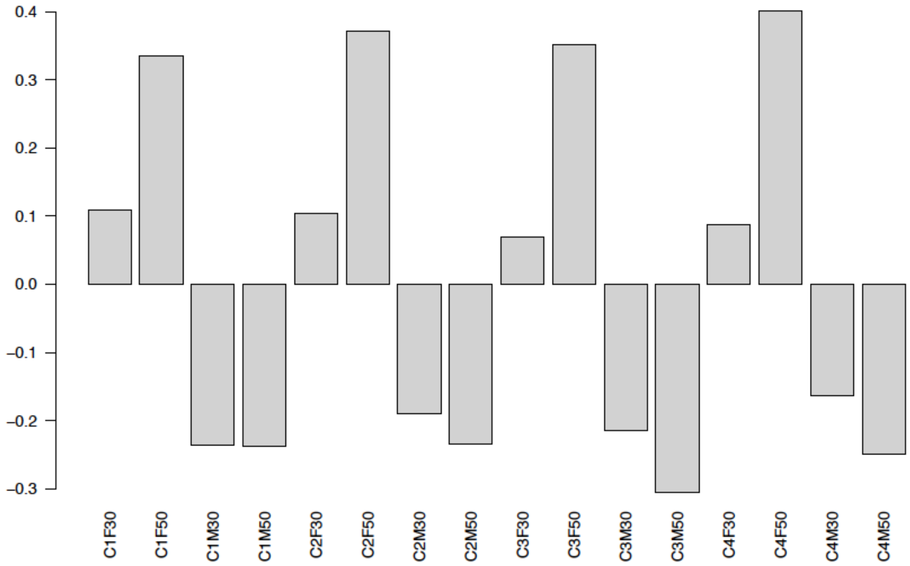

Supplemental Figure 15 - A: Time course relative abundances for phenylalanine. Left panel corresponds to mock treated samples, right corresponds to Fusarium graminearum treated samples., C1-C4 = NIL1-NIL4. B: Eigengene representation of the phenylalanine biosynthesis gene encoding prephenate dehydratase. C1-C4 = NIL1-4. M= mock, F= Fusarium, 30, 50 = 30 and 50 hai.
