# Supplementary material for: Joint Transcriptomic and Metabolomic Analyses Reveal Changes in the Primary Metabolism and Imbalances in the Subgenome Orchestration in the Bread Wheat Molecular Response to Fusarium graminearum
Source: G3 (Bethesda). 2015 Oct 4;5(12):2579–92. doi: 10.1534/g3.115.021550 (PMC4683631; doi:10.1534/g3.115.021550)
Supplement: Supporting Information [file supp_g3.115.021550_FigureS3.pdf]

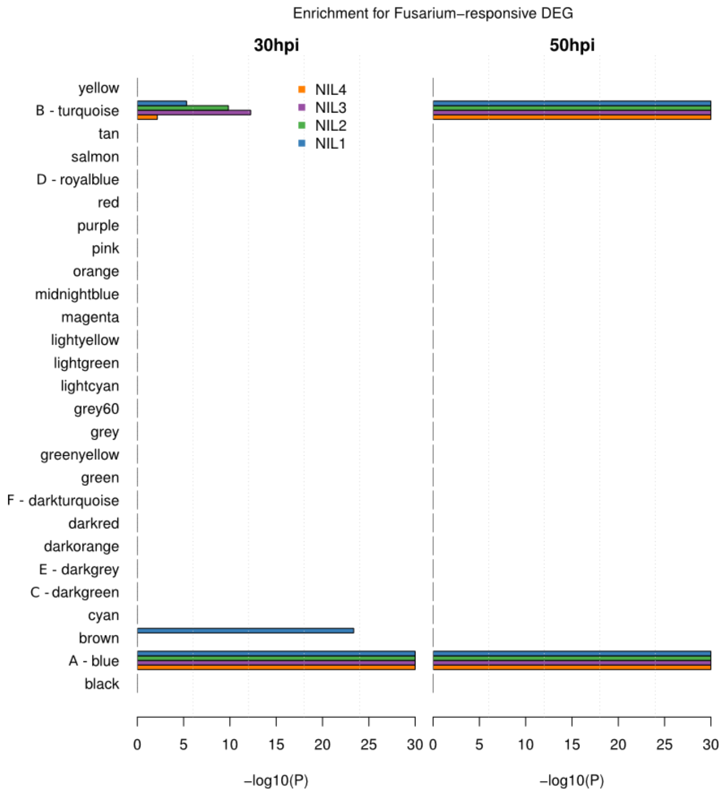

Supplemental Figure 3 - Enrichment for differentially expressed genes within the co-expression modules. FDR adjusted P values from a one-sided Fisher's exact test. For readability the bars were truncated at 10-30. Color names refer to the original WGCNA module labeling.
