# Supplementary material for: Joint Transcriptomic and Metabolomic Analyses Reveal Changes in the Primary Metabolism and Imbalances in the Subgenome Orchestration in the Bread Wheat Molecular Response to Fusarium graminearum
Source: G3 (Bethesda). 2015 Oct 4;5(12):2579–92. doi: 10.1534/g3.115.021550 (PMC4683631; doi:10.1534/g3.115.021550)
Supplement: Supporting Information [file supp_g3.115.021550_FigureS15.pdf]

A

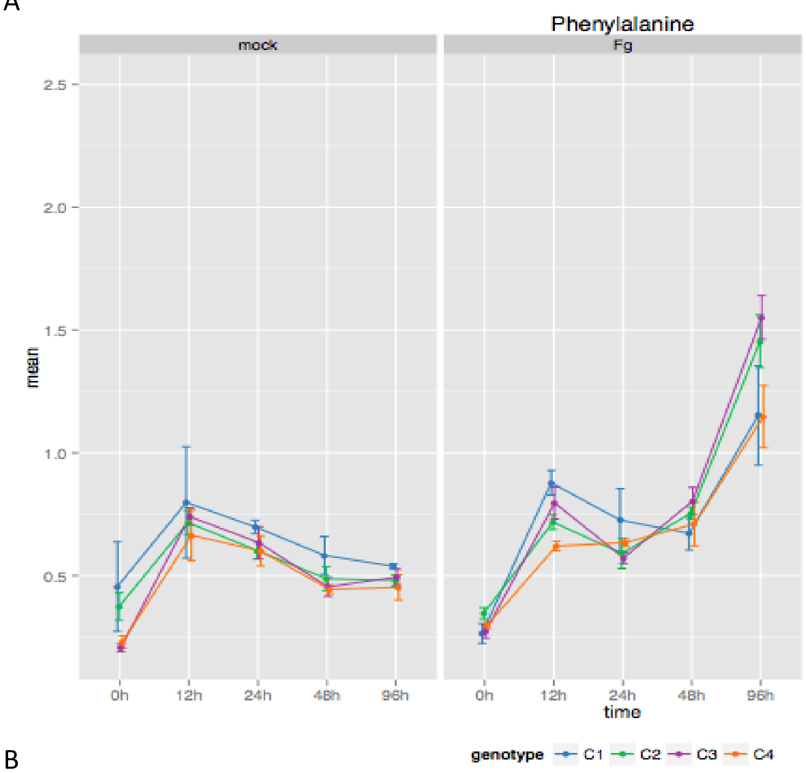

B

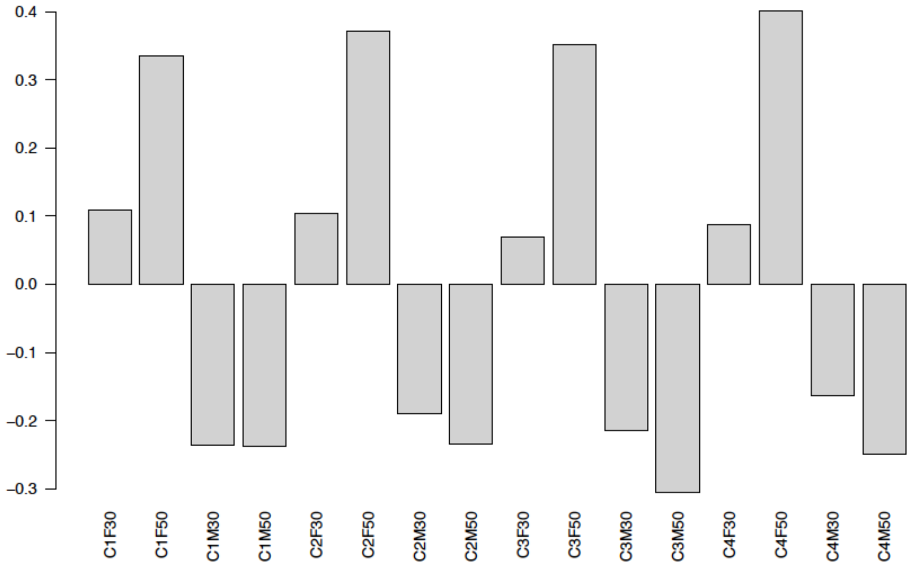

Supplemental Figure 15 - A: Time course relative abundances for phenylalanine. Left panel corresponds to mock treated samples, right corresponds to Fusarium graminearum treated samples., C1-C4 = NIL1-NIL4. B: Eigengene representation of the phenylalanine biosynthesis gene encoding prephenate dehydratase. C1-C4 = NIL1-4. M= mock, F= Fusarium, 30, 50 = 30 and 50 hai.
