# Supplementary material for: Joint Transcriptomic and Metabolomic Analyses Reveal Changes in the Primary Metabolism and Imbalances in the Subgenome Orchestration in the Bread Wheat Molecular Response to Fusarium graminearum
Source: G3 (Bethesda). 2015 Oct 4;5(12):2579–92. doi: 10.1534/g3.115.021550 (PMC4683631; doi:10.1534/g3.115.021550)
Supplement: Supporting Information [file supp_g3.115.021550_FigureS7.pdf]

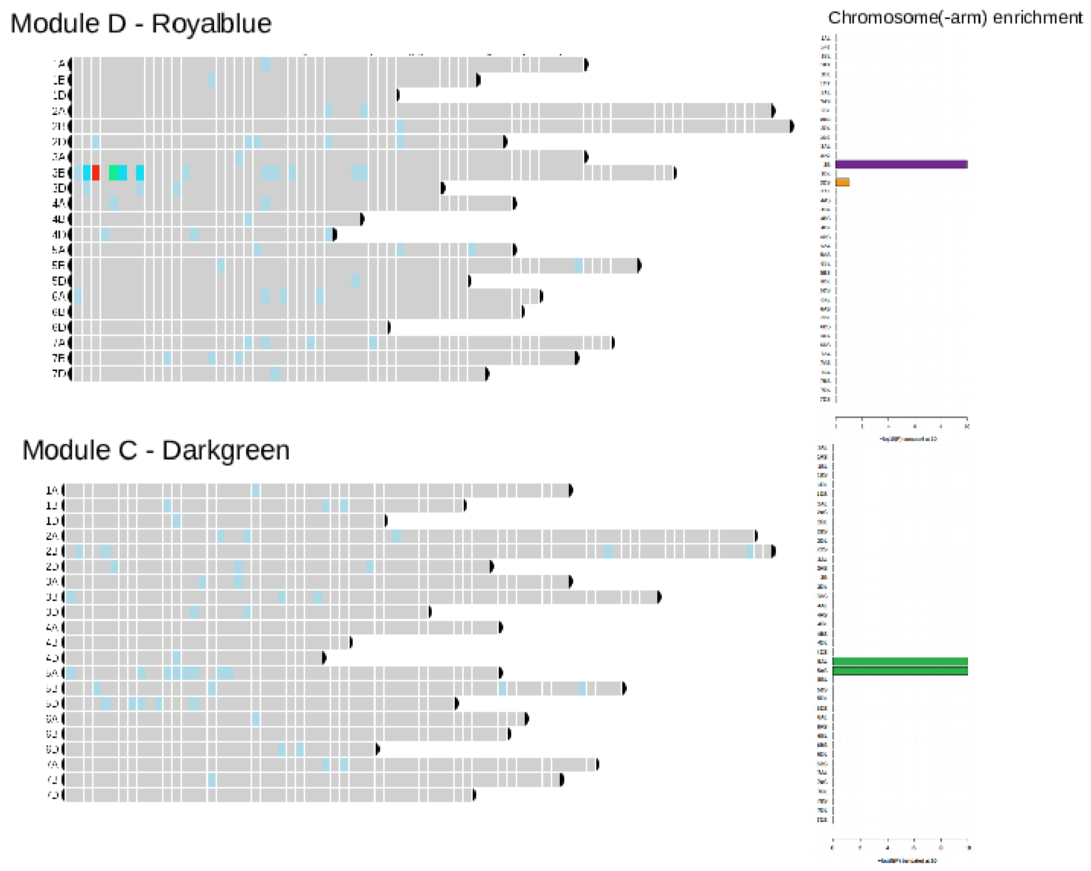

Supplemental Figure 7 - Visualization of the chromosomal positioning of the modules D (royalblue) and C (darkgreen) and the corresponding chromosome-arm enrichment with chromoWIZ (http://pgsb.helmholtz-muenchen.de/plant/chromoWIZ/). Color names refer to original WGCNA module names.
