# Supplementary figures and images for: Joint Transcriptomic and Metabolomic Analyses Reveal Changes in the Primary Metabolism and Imbalances in the Subgenome Orchestration in the Bread Wheat Molecular Response to Fusarium graminearum
Source: G3 (Bethesda). 2015 Oct 4;5(12):2579–92. doi: 10.1534/g3.115.021550 (PMC4683631; doi:10.1534/g3.115.021550)

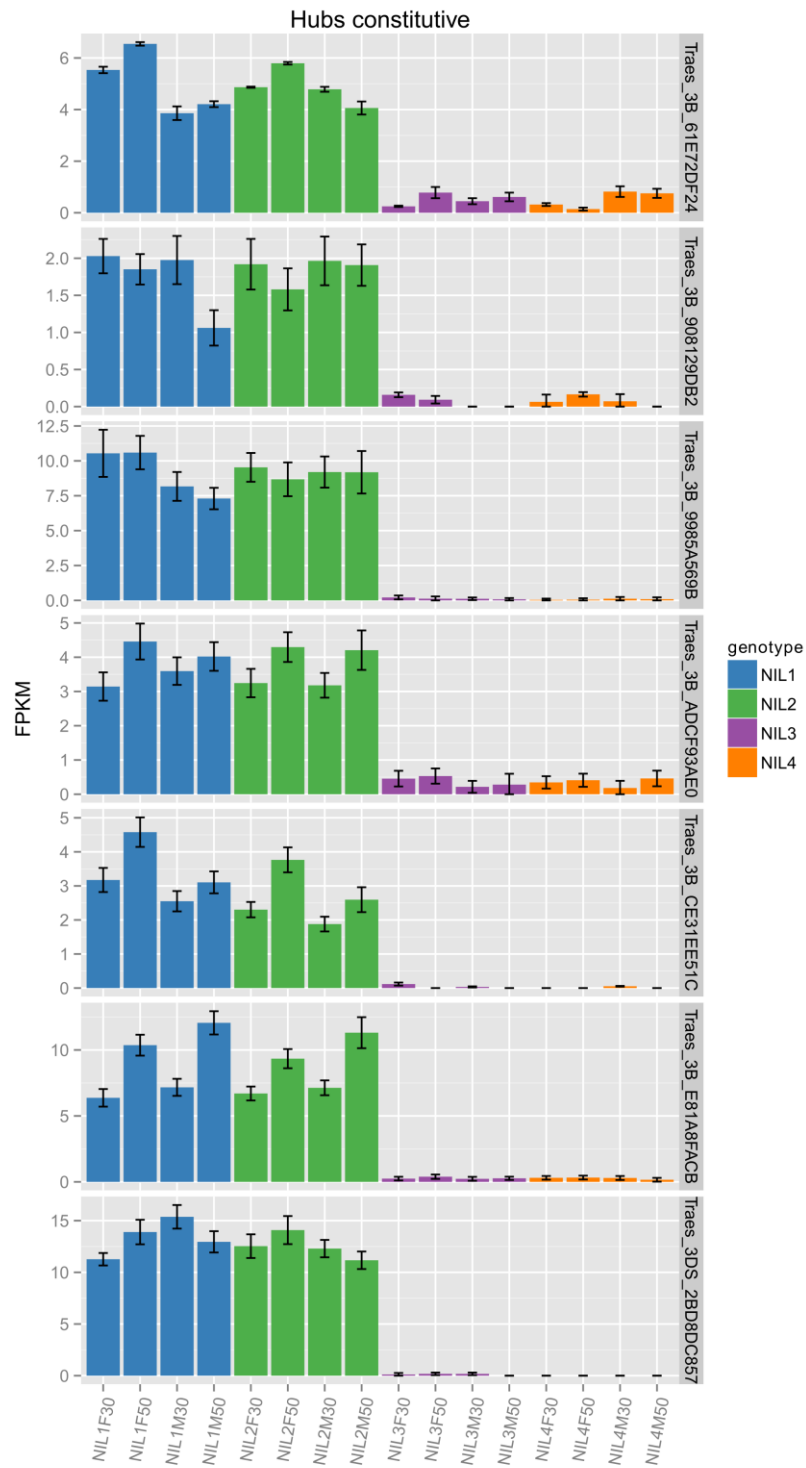

**Supplemental Figure 8** - Constitutive expression of hub genes on 3B and 3D.

Supplement: Supporting Information [file supp_g3.115.021550_FigureS8.pdf]
