# Supplementary material for: Joint Transcriptomic and Metabolomic Analyses Reveal Changes in the Primary Metabolism and Imbalances in the Subgenome Orchestration in the Bread Wheat Molecular Response to Fusarium graminearum
Source: G3 (Bethesda). 2015 Oct 4;5(12):2579–92. doi: 10.1534/g3.115.021550 (PMC4683631; doi:10.1534/g3.115.021550)
Supplement: Supporting Information [file supp_g3.115.021550_FigureS9.pdf]

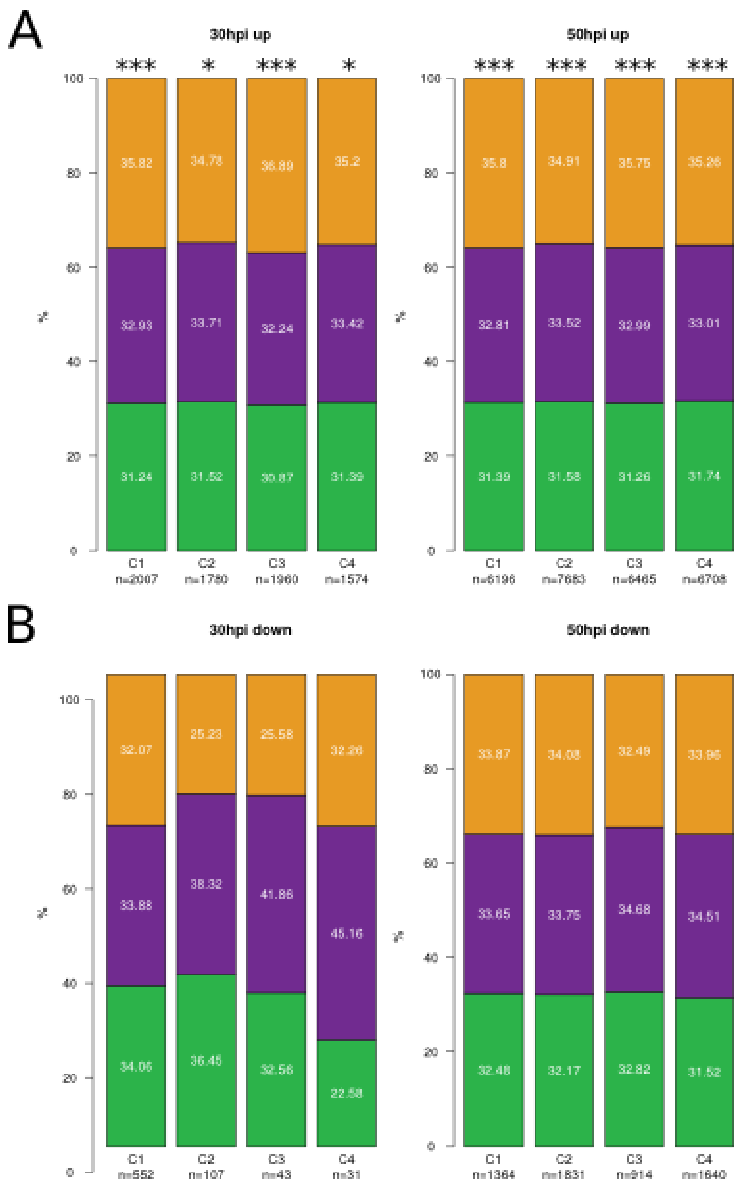

Supplemental Figure 9 - Subgenome-wise contribution to differentially expressed genes. (A) Genes that show increased expression after *Fusarium graminearum* treatment. (B) Genes that show reduced expression after *Fusarium graminearum* treatment.Significance of deviations from the expected distributions was quantified by a chi-squared test against 10,000 random multinomial distributions following the expected A, B, D subgenome distribution from the bread wheat high confidence gene set (* FDR adjusted P < 0.05; *** FDR adjusted P < 0.001). The number of genes with significant changes in expression levels is given as ‘n’.
