# Supplementary material for: Joint Transcriptomic and Metabolomic Analyses Reveal Changes in the Primary Metabolism and Imbalances in the Subgenome Orchestration in the Bread Wheat Molecular Response to Fusarium graminearum
Source: G3 (Bethesda). 2015 Oct 4;5(12):2579–92. doi: 10.1534/g3.115.021550 (PMC4683631; doi:10.1534/g3.115.021550)
Supplement: Supporting Information [file supp_g3.115.021550_FigureS11.pdf]

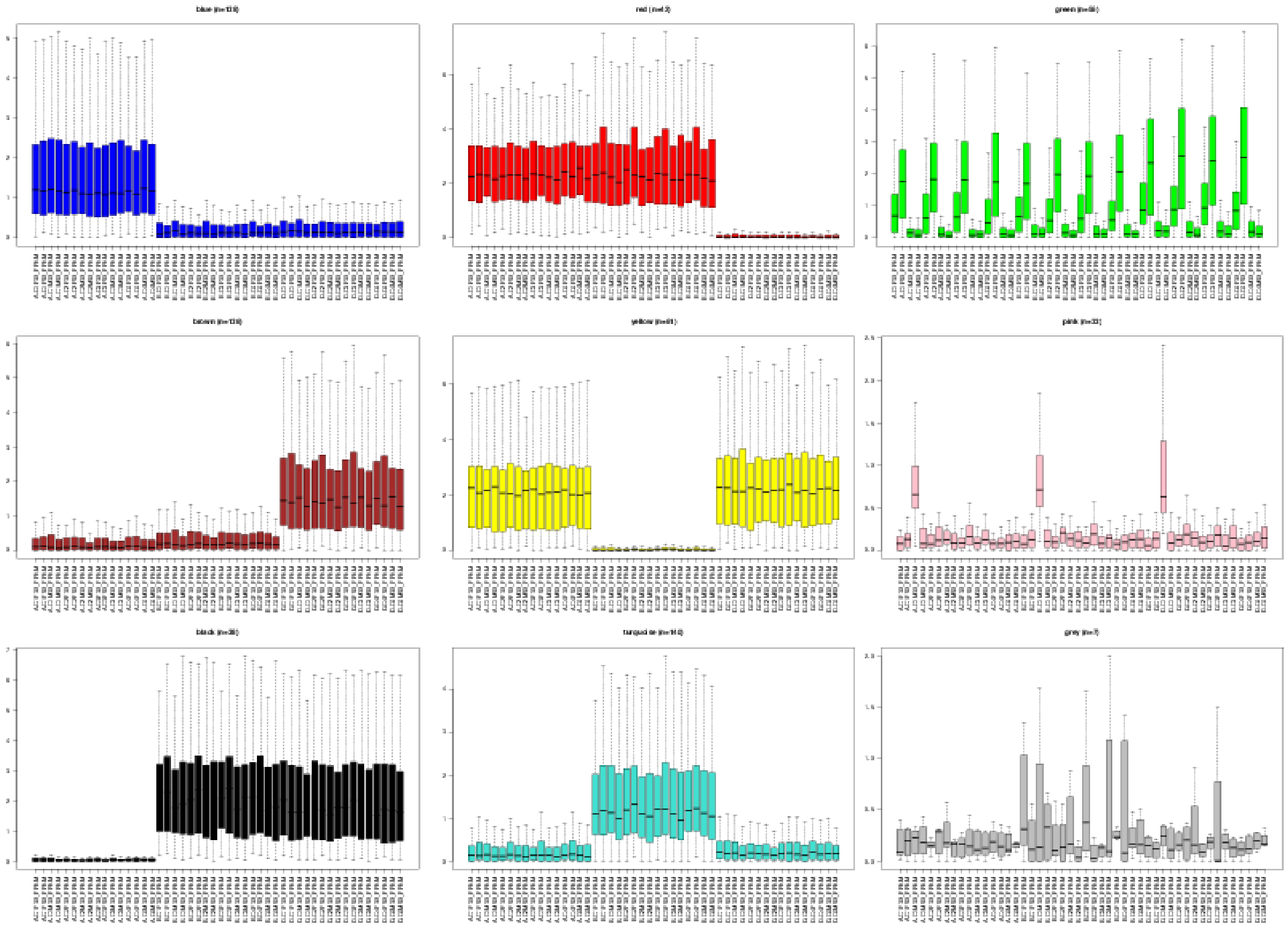

Supplemental Figure 11 - Module-wise expression in the triplet network. The first letter in the names on the x-axis indicates the subgenome (A, B, or D).
