# Supplementary material for: Joint Transcriptomic and Metabolomic Analyses Reveal Changes in the Primary Metabolism and Imbalances in the Subgenome Orchestration in the Bread Wheat Molecular Response to Fusarium graminearum
Source: G3 (Bethesda). 2015 Oct 4;5(12):2579–92. doi: 10.1534/g3.115.021550 (PMC4683631; doi:10.1534/g3.115.021550)
Supplement: Supporting Information [file supp_g3.115.021550_FigureS12.pdf]

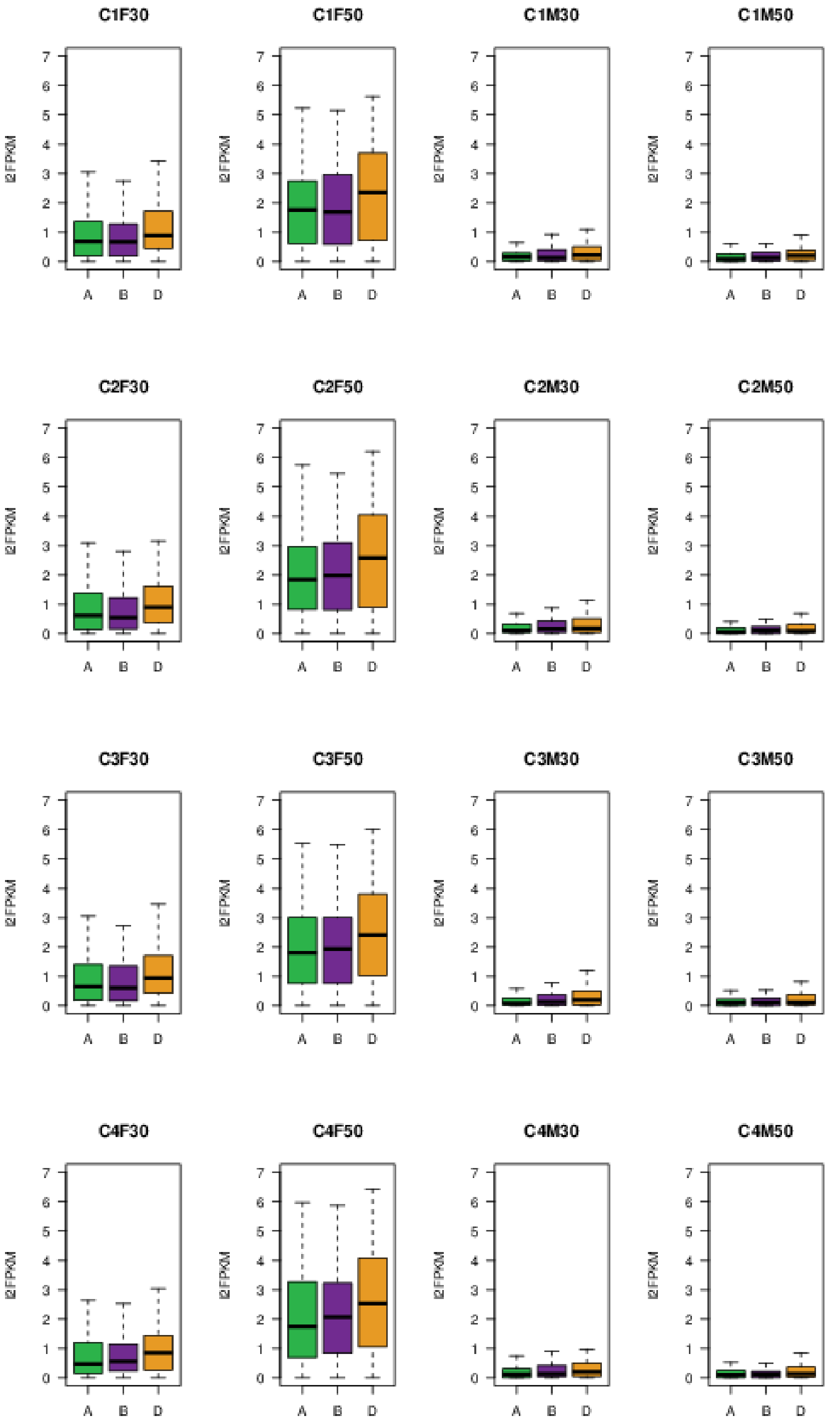


**Supplemental Figure 12 –** Condition-wise expression patterns for each of the four genotypes in the 'green' triplet network module. (F: *Fusarium graminearum* treatment; M: mock treatment; 30: 30hpi; 50: 50hpi)
