# Supplementary material for: Joint Transcriptomic and Metabolomic Analyses Reveal Changes in the Primary Metabolism and Imbalances in the Subgenome Orchestration in the Bread Wheat Molecular Response to Fusarium graminearum
Source: G3 (Bethesda). 2015 Oct 4;5(12):2579–92. doi: 10.1534/g3.115.021550 (PMC4683631; doi:10.1534/g3.115.021550)
Supplement: Supporting Information [file supp_g3.115.021550_FigureS13.pdf]

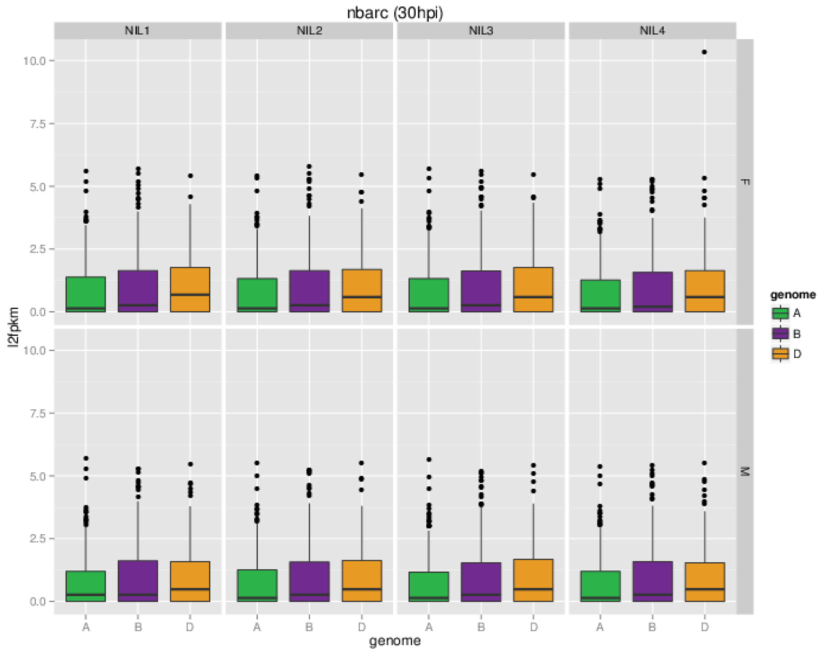

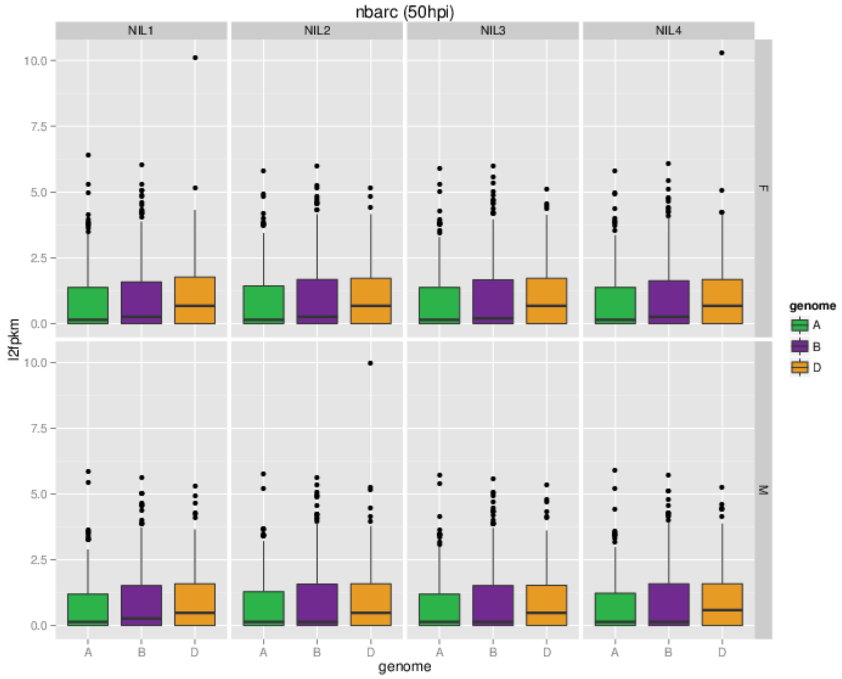

Supplemental Figure 13 – Expression of NB-ARC domain containing genes. Genes were extracted based on the NB-ARC Interpro domain (IPR002182; http://www.ebi.ac.uk/interpro/entry/IPR002182).
